# Preconception and Early-Pregnancy Body Mass Index in Women and Men, Time to Pregnancy, and Risk of Miscarriage

**DOI:** 10.1001/jamanetworkopen.2024.36157

**Published:** 2024-09-19

**Authors:** Aline J. Boxem, Sophia M. Blaauwendraad, Annemarie G. M. G. J. Mulders, Eline L. Bekkers, Claudia J. Kruithof, Eric A. P. Steegers, Romy Gaillard, Vincent W. V. Jaddoe

**Affiliations:** 1 The Generation R Study Group, Erasmus University Medical Centre, Rotterdam, the Netherlands; 2 Department of Paediatrics, Erasmus University Medical Centre, Rotterdam, the Netherlands; 3 Department of Obstetrics and Gynaecology, Erasmus University Medical Centre, Rotterdam, the Netherlands

## Abstract

**Importance:**

Obesity in women is associated with reduced fertility and an increased risk of miscarriage. These associations might also be present across the full range of body mass index (BMI) categories as well as among men.

**Objective:**

To investigate the associations of preconception BMI in both partners with time to pregnancy and miscarriage.

**Design, Setting, and Participants:**

This population-based prospective cohort study was conducted in Rotterdam, the Netherlands, between August 9, 2017, and July 1, 2021. A total of 3604 women and their partners were included from the preconception period onward with follow-up until birth. The date of analysis was July 12, 2024.

**Exposure:**

Body mass index (calculated as weight in kilograms divided by height in meters squared) measured in preconception or early pregnancy.

**Main Outcome and Measures:**

Fecundability, defined as the probability of conceiving within 1 month; subfertility, defined as time to pregnancy or duration of actively pursuing pregnancy of more than 12 months or use of assisted reproductive technology; and miscarriage, defined as pregnancy loss before 22 weeks of gestation. These measures were assessed using questionnaires and via the obstetric caregiver.

**Results:**

The study population for time-to-pregnancy analyses consisted of 3033 episodes among women (median age, 31.6 years [IQR, 29.2-34.5 years]; median BMI, 23.5 [IQR, 21.2-26.5]) and 2288 episodes among men (median age, 33.4 years [IQR, 30.5-36.8 years]; median BMI, 24.9 [IQR, 23.0-27.4]). The study population for miscarriage analyses consisted of 2770 pregnancy episodes among women (median age, 31.5 years [IQR, 28.9-34.3 years]; median BMI, 23.5 [IQR, 21.3-26.7]) and 2189 pregnancy episodes among men (median age, 33.5 years [IQR, 30.4-36.8 years]; median BMI, 25.0 [IQR, 23.0-27.5]). Higher BMI in women and men was associated with lower fecundability: for every unit increase in BMI, fecundability decreased (fecundability ratio [FR]: women, 0.98 [95% CI, 0.97-0.99]; men, 0.99 [95% CI, 0.98-1.00]). Women with overweight (FR, 0.88 [95% CI, 0.80-0.98]) and obesity (FR, 0.72 [95% CI, 0.63-0.82]) had lower fecundability compared with women with normal weight. Compared with normal weight in women, underweight (odds ratio [OR], 1.88 [95% CI, 1.22-2.88]), overweight (OR, 1.35 [95% CI, 1.11-1.63]), and obesity (OR, 1.67 [95% CI, 1.30-2.13]) were associated with increased odds of subfertility. In men, obesity was associated with increased odds of subfertility (OR, 1.69 [95% CI, 1.24-2.31]). Compared with normal weight in women, overweight (OR, 1.49 [95% CI, 1.12-1.98]) and obesity (OR, 1.44 [95% CI, 1.00-2.08]) were associated with increased odds of miscarriage.

**Conclusions and Relevance:**

In this cohort study, BMI outside of the normal category in women and men during the preconception or early-pregnancy periods was associated with time to pregnancy and miscarriage. Optimizing BMI in women and men from the preconception period onward might be an important strategy to improve fertility outcomes.

## Key Points

**Question:**

Is preconception body mass index (BMI) in both partners associated with time to pregnancy and risk of miscarriage?

**Findings:**

This prospective population-based cohort study including 3604 women showed that BMI outside of the normal category in women and men was associated with lower fecundability and subfertility. Overweight and obesity in women were associated with increased odds of miscarriage.

**Meaning:**

This study suggests that optimizing BMI in women and men from the preconception period onward might be an important strategy to improve fertility and reduce the risk of miscarriage.

## Teaser

This cohort study investigates the associations of preconception body mass index in both partners with time to pregnancy and risk of miscarriage.

## Introduction

Obesity among women of reproductive age is a major public health challenge.^1,2,3^ Obesity during pregnancy is associated with increased risks of gestational hypertensive disorders and diabetes, adverse birth outcomes, and cardiovascular disorders in their offspring throughout the life course.^3,4,5,6,7^ Obesity in women also seems to be associated with lower fertility, lower assisted reproductive technology success rates, and increased risks of miscarriage and pregnancy loss.^8,9,10,11,12,13,14,15,16,17,18^ Among subfertile individuals, overweight and obesity are associated with increased time until pregnancy is achieved.^8,11^ The association of body mass index (BMI; calculated as weight in kilograms divided by height in meters squared) with early-pregnancy outcomes might extend beyond the extremes of obesity.^19,20,21^ The optimum BMI in association with early-pregnancy outcomes is not known yet. A growing body of evidence among subfertile individuals suggests that next to BMI in women, BMI in men is also associated with fertility outcomes and the risk of miscarriage.^8,14,22,23,24,25^ A better understanding of the separate and combined associations of BMI in women and men with fertility and miscarriage outcomes is needed to develop novel targeted population strategies to optimize BMI from the preconception period onward.

We hypothesized that preconception BMI and early-pregnancy BMI outside of the normal category, not only in women but also in men, are associated with time to pregnancy and miscarriage. We assessed, in a population-based prospective cohort study from preconception onward among 3604 women and their partners, separate and combined associations of preconception BMI and early-pregnancy BMI in women and men with fecundability; subfertility, defined as a time to pregnancy or, in case of not conceiving, duration of actively pursuing pregnancy of more than 12 months or use of assisted reproductive technology; and miscarriage.

## Methods

### Study Design

This study was embedded in the Generation R *Next* Study, a population-based prospective cohort study from the preconception period onward in Rotterdam, the Netherlands, and is part of the Generation R Study Programme.^26^ The general aim of this study is to identify preconception and early-pregnancy determinants of fertility, embryonic development, and childhood outcomes. Women and their partners in the general population were eligible if they were 18 years of age or older, living in Rotterdam, and actively trying to conceive or were pregnant. Couples in preconception or pregnancy between August 9, 2017, and July 1, 2021, were included. Inclusion was aimed for those in preconception or early pregnancy but was allowed until delivery. In total, 33.2% of all inclusions (1339 of 4036) were in preconception and 52.8% (2129 of 4036) were in the first trimester. Study approval was obtained by the medical ethical committee of the Erasmus University Medical Centre, Rotterdam. Written informed consent was obtained from participating women and men. Findings were reported following the Strengthening the Reporting of Observational Studies in Epidemiology (STROBE) reporting guideline.^27^

Couples could participate more than once for different preconception or pregnancy episodes. In total, there were 4036 participant episodes from 3604 unique women, leading to 3577 pregnancies of 3200 unique women at the end of the study. In total, 71.3% of partners (2568 of 3604) participated in the study. For the present study, episodes without information on BMI (n = 264) were excluded. For the time-to-pregnancy analyses, we excluded episodes without information on time to pregnancy (n = 542) or, in case of not conceiving, duration of actively pursuing pregnancy (n = 197). A total of 3033 episodes (2856 unique women) were used. Fecundability analyses were based on 2739 episodes due to removing participants using assisted reproductive technology. Subfertility analyses were based on 3018 episodes due to removing participants with a duration of actively pursuing pregnancy of less than 12 months. For the miscarriage analyses, we excluded episodes without a pregnancy at the end of the study phase (n = 458), inclusion after 22 weeks of pregnancy (n = 316), or those withdrawn (n = 116), lost to follow-up (n = 35), or who moved outside the study area (n = 77). A total of 2770 pregnancy episodes (2507 unique women) were used for logistic regression analyses and 2763 pregnancy episodes for Cox proportional hazards regression analyses due to removing participants without a gestational age at miscarriage (eFigure 1 in Supplement 1).

### BMI in Women and Men

Height and weight of women were measured without shoes and heavy clothing at enrollment and subsequent preconception and/or first trimester visits. Height and weight of men were measured during the first trimester visit. If pregnant at enrollment, prepregnancy weight was obtained via questionnaires. Prepregnancy BMI per episode of women was computed by using prepregnancy weight as the basis and substituted with measurements at the 7-week (n = 229), 9-week (n = 97), or 11-week (n = 128) pregnancy visits if the prepregnancy weight of that episode was missing. We used only the first available BMI per episode, either preconception or early pregnancy. Body mass index was categorized as underweight (<18.5), normal weight (18.5-24.9), overweight (25.0-29.9), and obesity (≥30.0), as well as normal weight (18.5-24.9) and overweight and obesity (≥25.0).

### Time to Pregnancy and Miscarriage

Time to pregnancy and mode of conception were assessed through questionnaires in preconception and early pregnancy. We used questions regarding the date at which women started trying to conceive and refrained from using contraceptives (start date actively pursuing pregnancy) and the date of assisted reproductive technology, including intrauterine insemination, ovulation induction, in vitro fertilization (IVF), and intracytoplasmic sperm injection. First day of last menstrual period was obtained from the obstetric caregiver. In women who conceived, time to pregnancy was calculated from the start date of actively pursuing pregnancy and the first day of last menstrual period. In women who did not conceive, the duration of actively pursuing pregnancy was calculated from the start date of actively pursuing pregnancy and the last day of study participation or study end date. Fecundability was defined as the probability of conceiving within 1 month, defined as 28 days. Time to pregnancy was categorized into 2 groups: a time to pregnancy of 12 months or less (fertile) and a time to pregnancy or, in case of not conceiving, duration of actively pursuing pregnancy of more than 12 months or using assisted reproductive technology (subfertile).^28,29^ Spontaneous miscarriage was defined as pregnancy loss before 22 weeks of gestation.^30^ Date of miscarriage was obtained from the obstetric caregiver. Gestational age at miscarriage was based on the first day of last menstrual period, the due date based on ultrasonography, the date of intrauterine insemination, or embryo implant day minus 14 days in case of IVF or intracytoplasmic sperm injection.

### Covariates

Information on age, ethnicity, and highest educational level was obtained through questionnaires at enrollment. This was a multiethnic study. Ethnicity is strongly associated with both the exposure and outcomes of interest.^12,31^ Ethnicity (Dutch, other non-Western [African; American, non-Western; Asian, non-Western; Chinese; or Indonesian], and other Western [American, Western; Asian, Western; Cape Verdean; Dutch Antilles; European; German; Yugoslav; Moroccan; Oceanian; Polish; Surinamese; or Turkish]) was based on questions regarding the birth country of the participants and their parents and classified according to Statistics Netherlands.^32,33^ (Statistics Netherlands defines a migration background as Western or non-Western because socioeconomic position and sociocultural values from non-Western countries differ from those of most populations in industrialized Western countries.) If one of the participants’ parents was born abroad, participants were classified as of non-Dutch ethnic origin. If both parents were born abroad, the birth country of the mother defined the participants’ ethnicity. Information on smoking, alcohol consumption, parity, and history of previous miscarriage was assessed through questionnaires answered during preconception and/or early pregnancy.

### Statistical Analysis

First, we performed a follow-up nonresponse analysis comparing characteristics of women and men with and women and men without information on time to pregnancy or miscarriage by using the *t* test, the Mann-Whitney *U* test, the χ^2^ test, or the Fisher exact test. Second, we examined the associations of BMI in women and men separately with fecundability and miscarriage using Cox proportional hazards regression models (R package survival; R, version 4.2.3 [R Project for Statistical Computing]). Survival outcomes were conception or no conception and miscarriage or no miscarriage, respectively. The time variable for fecundability was based on time to pregnancy or duration of actively pursuing pregnancy in months, defined as 28 days. Women who underwent assisted conception were excluded from the fecundability analysis due to unknown time to pregnancy. The time variable for miscarriage was based on gestational age in weeks. We checked the proportional hazards assumptions of the covariates using Schoenfeld residuals, assessed linearity of all associations using Martingale residuals, and assessed influentials using deviance residuals. The resulting hazard ratio from the Cox proportional hazards regression model represents the fecundability ratio (FR; eMethods in Supplement 1), and the probability of miscarrying per week of gestation, compared with the mean BMI or reference category. Third, we examined the associations of BMI in women and men separately with the odds of subfertility and miscarriage using logistic regression models. For these analyses, BMI was analyzed as a continuous and categorical variable. To assess the independent associations of BMI in women and men with the outcomes, we repeated the analyses and constructed a third model in which we mutually adjusted for BMI of women and men. To assess cumulative associations with the outcomes, we performed regression analyses with combinations of BMI categories for episodes in which both women and men participated (normal weight in both partners, overweight and obesity in women only, overweight and obesity in men only, and overweight and obesity in both partners). The statistical interaction term between BMI of women and BMI of men was not statistically significant. These regression models were first analyzed in univariate models and second in multivariate models with adjustment for potential confounders. Potential confounders were selected a priori based on a directed acyclic graph and existing literature (eFigures 2 and 3 in Supplement 1) and included age, ethnicity, educational level, smoking, alcohol consumption, parity, and history of previous miscarriage.

We performed 3 sensitivity analyses. First, because some women participated more than once, we repeated the analyses by including only their first study episode. Second, to assess whether any association was influenced by assisted reproductive technology, we excluded participants who underwent assisted conception. Although we have information about assisted reproductive technology, we do not have information about specific indications in women or men. Third, to account for the right-skewed time-to-pregnancy distribution, the Cox proportional hazards regression analysis was repeated excluding the top 5% of the time-to-pregnancy distribution. Missing values were imputed using multiple imputation by chained equations to reduce potential bias due to missing values of covariates (R package mice). Pooled results were reported. Analyses were performed using R Statistical Software, version 4.2.3 (R Project for Statistical Computing). All *P* values were from 2-sided tests and results were deemed statistically significant at *P* < .05.

## Results

### Population Characteristics

The Table shows characteristics for time-to-pregnancy and miscarriage populations. The study population for time-to-pregnancy analyses consisted of 3033 episodes among women (median [IQR] age, 31.6 years [IQR, 29.2-34.5 years]; 1875 Dutch [62.1%]; median BMI, 23.5 [IQR, 21.2-26.5]) and 2288 episodes among men (median age, 33.4 years [IQR, 30.5-36.8 years]; 1506 Dutch [66.3%]; median BMI, 24.9 [IQR, 23.0-27.4]). The study population for miscarriage analyses consisted of 2770 pregnancy episodes among women (median age, 31.5 years [IQR, 28.9-34.3 years]; 1620 Dutch [63.2%]; median BMI, 23.5 [IQR, 21.3-26.7]) and 2189 pregnancy episodes among men (median age, 33.5 years [IQR, 30.4-36.8 years]; 1370 Dutch [66.1%]; median BMI, 25.0 [IQR, 23.0-27.5]). The median time to pregnancy was 3.7 months (95% range [2.5%-97.5%], 0.0-68.3 months). In total, 541 of the 3033 episodes (17.8%) were subfertile, and 314 of the 2770 pregnancy episodes (11.3%) led to a miscarriage. Prepregnancy weight was not available in 383 of the 2770 episodes, for which we used the first early-pregnancy weight measurement. eTables 1 and 2 in Supplement 1 show characteristics of participants and episodes with or without an observed outcome. Nonresponse analyses showed a loss to follow-up of 7.8% (296 of 3772) when comparing women with and women without an observed outcome (eTables 1 and 2 in Supplement 1). Women and men included in the study populations were older, had a lower BMI, and were more frequently Dutch compared with those not included. The percentage of missing data for covariates ranged from 0% to 18.4% (509 of 2770).

**Table.** Characteristics of Episodes of Included Participants^a^

Characteristic	Episodes, No. (%)				
	Time to pregnancy		Miscarriage	
	Women (n = 3033)^b^	Men (n = 2288)	Women (n = 2770)^c^	Men (n = 2189)
Age at enrollment, median (IQR), y	31.6 (29.2-34.5)	33.4 (30.5-36.8)	31.5 (28.9-34.3)	33.5 (30.4-36.8)
<30 y	983 (32.4)	495 (21.7)	952 (34.4)	498 (22.8)
30-35 y	1382 (45.6)	944 (41.3)	1258 (45.5)	875 (40.0)
>35 y	668 (22.0)	847 (37.1)	560 (20.2)	814 (37.2)
Missing	0	2 (0.1)	0	2 (0.1)
Ethnicity				
Dutch	1875 (62.1)	1506 (66.3)	1620 (63.2)	1370 (66.1)
Other non-Western^d^	268 (8.9)	208 (9.2)	224 (8.7)	193 (9.3)
Other Western^e^	874 (29.0)	558 (24.6)	719 (28.1)	511 (24.6)
Missing	16 (0.5)	16 (0.7)	207 (7.5)	115 (5.3)
Educational level				
No education finished, primary education finished, or secondary education finished	847 (28.3)	778 (34.2)	721 (28.3)	713 (34.3)
Higher education finished	2151 (71.7)	1497 (65.8)	1823 (71.7)	1365 (65.7)
Missing	35 (1.2)	13 (0.6)	226 (8.2)	111 (5.1)
BMI, median (IQR)	23.5 (21.2-26.5)	24.9 (23.0-27.4)	23.5 (21.3-26.7)	25.0 (23.0-27.5)
Underweight (<18.5)	97 (3.2)	17 (0.7)	71 (2.6)	17 (0.8)
Normal weight (18.5-24.9)	1860 (61.3)	1141 (49.9)	1688 (60.9)	1081 (49.4)
Overweight (25-29.9)	711 (23.4)	881 (38.5)	655 (23.6)	838 (38.3)
Obesity (≥30)	365 (12.0)	249 (10.9)	356 (12.9)	253 (11.6)
Missing	0	0	0	0
Smoking				
No	1568 (55.4)	1148 (50.9)	1249 (55.2)	1014 (50.7)
Quit smoking before pregnancy	886 (31.3)	705 (31.3)	679 (30.0)	634 (31.7)
Smoked during pregnancy	375 (13.3)	401 (17.8)	333 (14.7)	352 (17.6)
Missing	204 (6.7)	34 (1.5)	509 (18.4)	189 (8.6)
Alcohol consumption				
No consumption <3 mo before pregnancy	619 (21.0)	266 (11.8)	527 (21.6)	227 (11.4)
Consumption <3 mo before pregnancy	1905 (64.6)	1984 (88.2)	1502 (61.5)	1771 (88.6)
Consumption during pregnancy	427 (14.5)	NA	412 (16.9)	NA
Missing	82 (2.7)	38 (1.7)	329 (11.9)	191 (8.7)
Parity				
Nulliparous	1945 (65.7)	1509 (66.9)^f^	1575 (63.9)	1358 (66.7)
Multiparous	1014 (34.3)	745 (33.1)^f^	891 (36.1)	678 (33.3)
Missing	74 (2.4)	34 (1.5)^f^	304 (11.0)	153 (7.0)
Miscarriage in previous pregnancy				
No	2227 (79.2)	1697 (80.7)^f^	1780 (79.8)	1496 (81.0)^f^
Yes	584 (20.8)	406 (19.3)^f^	450 (20.2)	352 (19.0)^f^
Missing	222 (7.3)	185 (8.1)^f^	540 (19.5)	341 (15.6)^f^
Time to pregnancy, median (95% range [2.5%-97.5%]), mo^g^	3.7 (0.0-68.3)	3.3 (0.0-59.5)^f^	3.6 (0.0-65.3)	3.3 (0.0-56.6)^f^
≤12 mo	1944 (64.1)	1692 (74.0)^f^	1647 (70.2)	1459 (74.3)^f^
>12 mo^h^	541 (17.8)	382 (16.7)^f^	435 (18.5)	319 (16.2)^f^
ART leading to pregnancy^h^	287 (9.5)	214 (9.4)^f^	245 (10.5)	186 (9.5)^f^
Not pregnant^h^	261 (8.6)	0^f^	NA	0^f^
Missing	0	0^f^	443 (16.0)	225 (10.3)^f^
Occurrence of miscarriage				
Miscarriage	219 (7.9)	135 (5.9)^f^	314 (11.3)	174 (7.9)^f^
No miscarriage	2553 (92.1)	2153 (94.1)^f^	2456 (88.7)	2015 (92.1)^f^
Missing	261 (8.6)	0^f^	0	0^f^
Timing of miscarriage, median (IQR), wk	8.1 (7.0-9.4)	8.6 (7.6-9.6)^f^	8.3 (7.1-9.5)	8.6 (7.9-9.6)^f^
First trimester	201 (92.6)	122 (91.0)^f^	284 (92.2)	158 (91.9)^f^
Second trimester	16 (7.4)	12 (9.0)^f^	24 (7.8)	14 (8.1)^f^
Missing	2 (0.9)	1 (0.7)^f^	6 (1.9)	2 (1.1)^f^

Abbreviations: ART, assisted reproductive technology; BMI, body mass index (calculated as weight in kilograms divided by height in meters squared); NA, not applicable.

^a^ Total study population consisting of 3604 unique women from Rotterdam, the Netherlands, with a total of 4036 participant episodes, leading to 3577 pregnancy episodes. Women were included in preconception and pregnancy between 2017 and 2021.

^b^ Study population of time to pregnancy consisting of 2856 unique women from Rotterdam, the Netherlands, with a total of 3033 episodes.

^c^ Study population of miscarriage consisting of 2507 unique women with a total of 2770 pregnancy episodes.

^d^ Included African; American, non-Western; Asian, non-Western; Chinese; or Indonesian.

^e^ Included American, Western; Asian, Western; Cape Verdean; Dutch Antilles; European; German; Yugoslav; Moroccan; Oceanian; Polish; Surinamese; or Turkish.

^f^ Parity, miscarriage in previous pregnancy, time to pregnancy in months, occurrence of miscarriage, and timing of miscarriage in weeks in men were derived from their partner.

^g^ Time to pregnancy in months was derived from pregnancy episodes with a natural conception.

^h^ Episodes with ART leading to pregnancy (n = 287), episodes without pregnancy and use of ART (7 of 261 episodes), and episodes without pregnancy and duration of actively pursuing pregnancy of more than 12 months (218 of 261 episodes) were added to the subfertile group (time to pregnancy >12 months and use of ART) in the analysis.

### Fecundability and Subfertility

**Figure 1.**
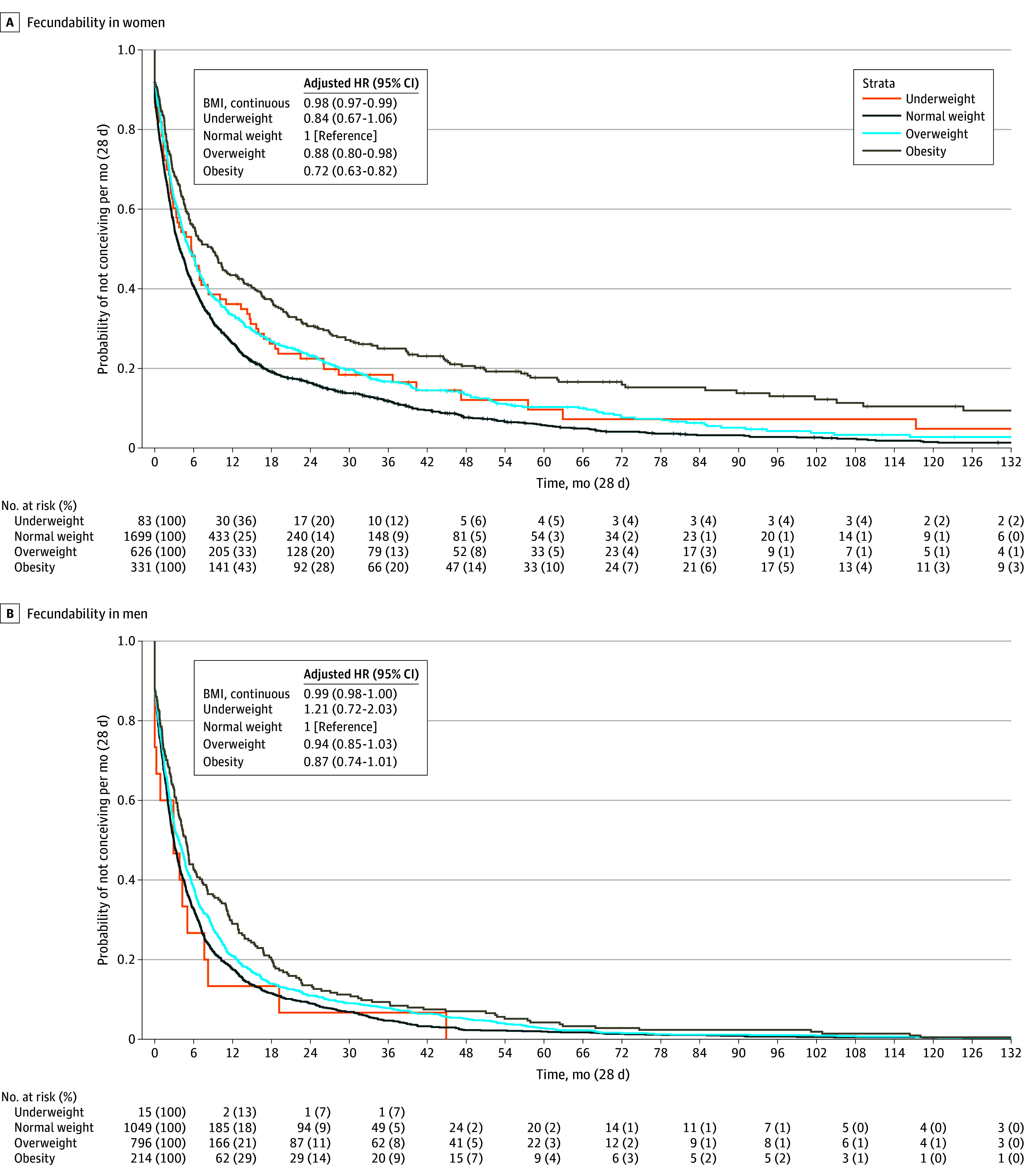
Associations of Body Mass Index (BMI) in Women and Men With Their Adjusted Fecundability Ratios Fecundability ratios (with 95% CIs) associated with BMI (calculated as weight in kilograms divided by height in meters squared) categories. Fecundability ratios were derived from the hazard ratios (HRs) of the Cox proportional hazards regression model. The fecundability ratio of BMI on the categorical scale was calculated as follows: HR = hazard rate [*H*(*t*)] of the different BMI categories / [*H*(*t*) BMI reference category (normal weight)]. An HR less than 1 indicates a lower fecundability compared with the reference category. A, Main analysis for women with adjustment for participant’s age, ethnicity, educational level, smoking, alcohol consumption, and parity. The survival curve was derived from the unadjusted model. B, Main analysis for men with adjustment for participant’s age, ethnicity, educational level, smoking, and alcohol consumption. The survival curve was derived from the unadjusted model. Several sensitivity analyses were performed excluding the top 5% observations of time to pregnancy correcting for the right-skewed distribution (eTable 4 in Supplement 1), including only first episodes of participants (eTable 5 in Supplement 1), and including partner’s BMI as a covariate (eTable 6 in Supplement 1).

Figure 1 shows that for every unit increase in BMI in women and men, fecundability decreased (FR: women, 0.98 [95% CI, 0.97-0.99]; men, 0.99 [95% CI, 0.98-1.00]). Compared with women with normal weight, those with overweight (FR, 0.88 [95% CI, 0.80-0.98]) and obesity (FR, 0.72 [95% CI, 0.63-0.82]) had a lower fecundability (eTables 3 and 4 in Supplement 1). Analyses on combined BMI categories in both partners showed that compared with normal weight in both partners, overweight and obesity in men only decreased fecundability (FR, 0.89 [95% CI, 0.80-1.00]) (eTable 8 in Supplement 1).

**Figure 2.**
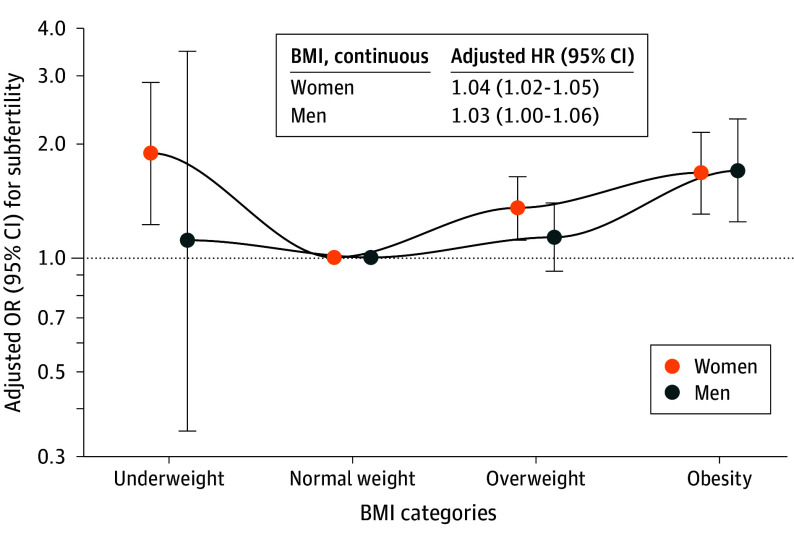
Associations of Body Mass Index (BMI) in Women and Men With Adjusted Odds Ratios (ORs) of Subfertility Adjusted ORs (with 95% CIs) of subfertility (time to pregnancy or duration of actively pursuing pregnancy >12 months and assisted reproductive technology) associated with BMI categories. Odds ratios were derived from the exponentiated coefficients of the logistic regression models. An OR greater than 1 indicates increased odds of subfertility compared with the reference category (normal weight). Main analysis for women was adjusted for participant’s age, ethnicity, educational level, smoking, alcohol consumption, and parity. Main analysis for men was adjusted for participant’s age, ethnicity, educational level, smoking, and alcohol consumption (OR of underweight in men must be interpreted with caution due to the small sample size in this group). Several sensitivity analyses were performed, excluding episodes with assisted reproductive technology (eTable 11 in Supplement 1), excluding the top 5% observations of time to pregnancy correcting for the right-skewed distribution (eTable 12 in Supplement 1), including only first episodes of participants (eTable 13 in Supplement 1), and including partner’s BMI as a covariate (eTable 14 in Supplement 1).

Higher BMI in women and men was associated with subfertility (odds ratio [OR]: women, 1.04 [95% CI, 1.02-1.05]; men, 1.03 [95% CI, 1.00-1.06]) (Figure 2). Compared with women with normal weight, women with underweight (OR, 1.88 [95% CI, 1.22-2.88]), overweight (OR, 1.35 [95% CI, 1.11-1.63]), and obesity (OR, 1.67 [95% CI, 1.30-2.13]) had increased odds of subfertility (Figure 2; eTables 9 and 10 in Supplement 1). Among men, obesity was associated with subfertility (OR, 1.69 [95% CI, 1.24-2.31]). Analyses on combined BMI categories of both partners showed that compared with normal weight in both partners, overweight and obesity in both partners was associated with subfertility (OR, 1.41 [95% CI, 1.06-1.87]) (eTable 15 in Supplement 1). Sensitivity analyses excluding women who underwent assisted reproductive technology, excluding women in the top 5% of time-to-pregnancy observations, restricting analyses to only participants’ first episode, or adjusting for partners’ BMI did not materially change the effect estimates (eTables 5-7 and eTables 11-14 in Supplement 1).

### Miscarriage

**Figure 3.**
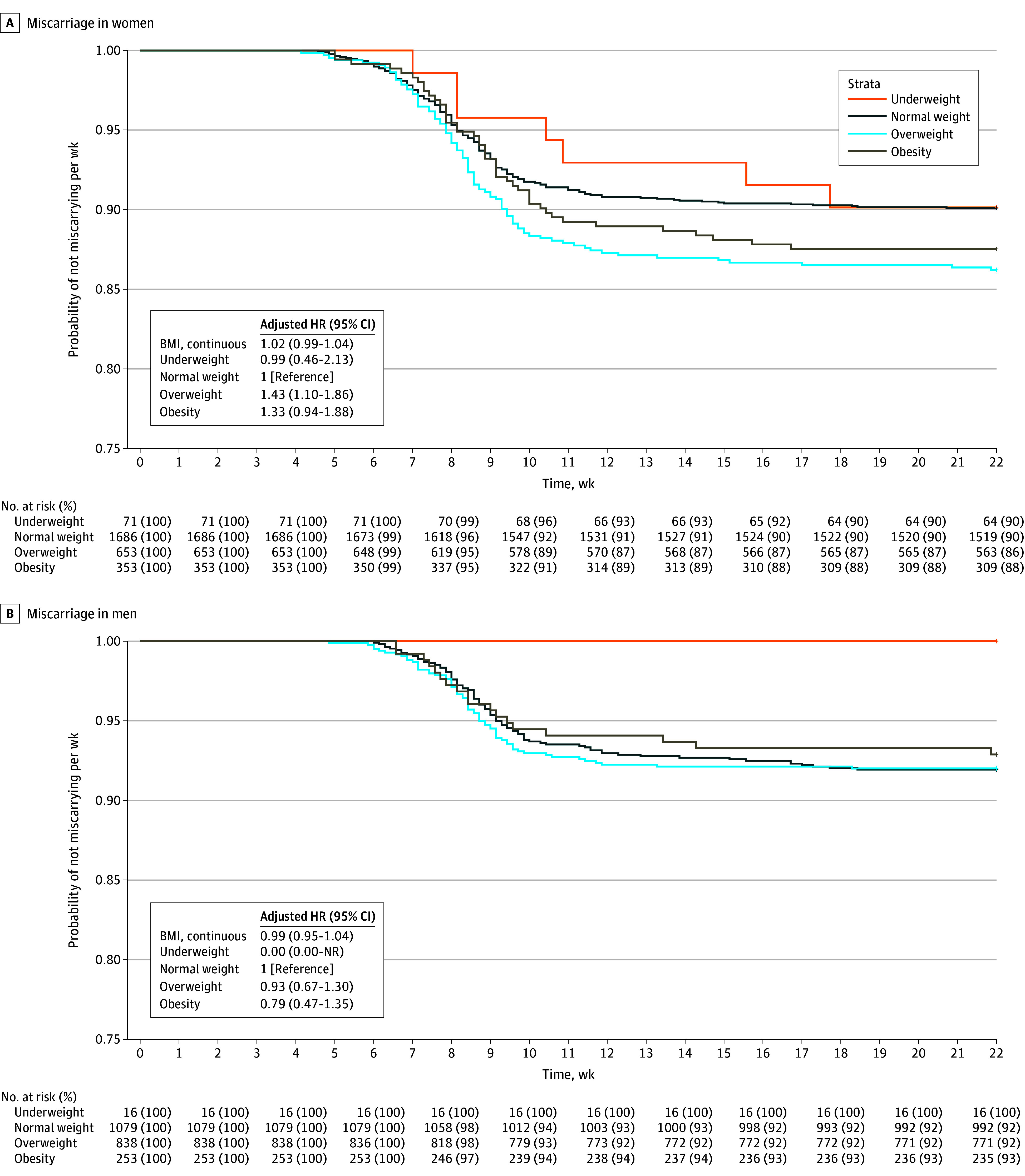
Associations of Body Mass Index (BMI) in Women and Men With Their Adjusted Hazard Ratios (HRs) for Miscarriage Adjusted HRs (with 95% CIs) of miscarriage associated with BMI categories. Hazard ratios were derived from the Cox proportional hazards regression model. The HR of BMI on the categorical scale was calculated as follows: HR = hazard rate [*H*(*t*)] of the different BMI categories / [*H*(*t*) BMI reference category (normal weight)]. An HR greater than 1 indicates a higher probability of miscarriage per week compared with the reference category. A, Main analysis for women with adjustment for participant’s age, ethnicity, educational level, smoking, alcohol consumption, parity, and history of miscarriage. The survival curve was derived from the unadjusted model. B, Main analysis for men with adjustment for participant’s age, ethnicity, educational level, smoking, and alcohol consumption. The survival curve was derived from the unadjusted model. Several sensitivity analyses were performed, excluding episodes with assisted reproductive technology (eTable 18 in Supplement 1), including only first episodes of participants (eTable 19 in Supplement 1), and including partner’s BMI as a covariate (eTable 20 in Supplement 1). NR indicates not reached.

**Figure 4.**
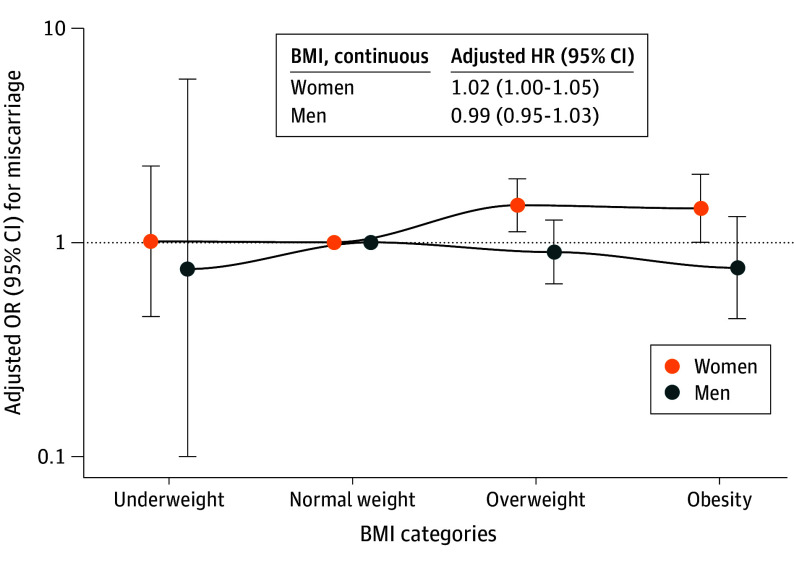
Associations of Body Mass Index (BMI) in Women and Men and Adjusted Odds Ratios (ORs) of Miscarriage Adjusted ORs (with 95% CIs) of miscarriage associated with BMI categories. Odds ratios were derived from the exponentiated coefficients of the logistic regression models. An OR greater than 1 indicates increased odds of miscarriage compared with the reference category (normal weight). Main analysis for women was adjusted for participant’s age, ethnicity, educational level, smoking, alcohol consumption, parity, and history of miscarriage. Main analysis for men was adjusted for participant’s age, ethnicity, educational level, smoking, and alcohol consumption (OR of underweight in men must be interpreted with caution due to the small sample size in this group). Several sensitivity analyses were performed, excluding episodes with assisted reproductive technology (eTable 24 in Supplement 1), including only first episodes of participants (eTable 25 in Supplement 1), and including partner’s BMI as a covariate (eTable 26 in Supplement 1).

Compared with normal weight in women, overweight increased the probability of miscarriage per week (hazard ratio, 1.43 [95% CI, 1.10-1.86]) (Figure 3). This finding might be explained by the small number of women with underweight and obesity in our study population. We did not observe other associations of BMI in women and men with the probability of miscarriage per week (eTables 16-17 and eTable 21 in Supplement 1). We did not observe associations of BMI on the continuous scale in women and men with the odds of miscarriage (Figure 4). Compared with normal weight in women, overweight (OR, 1.49 [95% CI, 1.12-1.98]) and obesity (OR, 1.44 [95% CI, 1.00-2.08]) were associated with increased odds of miscarriage (Figure 4; eTables 22 and 23 in Supplement 1). We did not observe associations of BMI in men with miscarriage (eTables 22 and 23 in Supplement 1). Analyses on combined BMI categories in both partners showed no associations (eTable 27 in Supplement 1). Sensitivity analyses excluding women using assisted reproductive technology, restricting analyses to only participants’ first episode, or adjusting for partners’ BMI did not materially change the effect estimates (eTables 18-20 and eTables 24-26 in Supplement 1).

## Discussion

In this population-based prospective cohort study from the preconception period onward, we observed that preconception BMI across the full range in women and men was associated with lower fecundability and subfertility. Overweight and obesity in women before or during early pregnancy was associated with increased odds of miscarriage.

Obesity among women of reproductive age is a well-known risk factor for subfertility, pregnancy complications, and impaired cardiovascular health in offspring. The associations of BMI with early-pregnancy outcomes might extend beyond the extremes of obesity, and in both women and men.^2,3,4,5^ Optimizing BMI might be an important population health strategy to improve fertility and early-pregnancy outcomes. Therefore, we assessed whether preconception BMI across the full range in women and men is associated with time to pregnancy and miscarriage.

We observed that BMI outside of the normal category in women was associated with lower fecundability and subfertility, even when excluding couples undergoing assisted reproductive technology. A retrospective cohort study among 84 075 women in Norway reported associations between a higher BMI in women and lower fecundability.^18^ Most other studies were conducted among subfertile individuals or focused on overweight and obesity only.^8,11,17,18,19,31,34,35,36,37,38^ We also observed an association of underweight in women with subfertility. This association has been previously reported in studies among nulliparous women or with retrospective designs.^19,34^ We observed that higher BMI in men was associated with lower fecundability. A retrospective cohort study among 68 002 men in Norway reported associations between higher BMI in men and lower fecundability.^18^ Retrospective cohort studies among 50 927 couples in China and 2112 men in the UK did not report associations between BMI in men and lower fecundability.^37,39^ Another retrospective cohort study among 2 301 782 couples in China reported associations of underweight in men with subfertility.^34^ A prospective cohort study among 501 couples in the US did not observe associations of female or male BMI with fecundability but reported that severe obesity in couples decreased fecundability.^40^ These inconsistent findings may be explained by the characteristics of the underlying study population. When we combined the BMI of women and men in 1 model, the associations of higher BMI in men with fecundability was independent of BMI in women. We observed the strongest associations with subfertility when overweight and obesity was present in both partners. This finding emphasizes the potential independent negative association of male BMI with fertility, possibly owing to lower sperm quality.^8,24,41^ The associations of overweight and obesity in women only and in both partners with fecundability and the associations of overweight and obesity in women or men only with subfertility were of borderline significance, possibly due to lower numbers in these groups. Thus, in this large European population-based cohort, BMI across the full range in women and men was associated with lower fecundability and subfertility.

Obesity in women is a known risk factor of miscarriage.^10,17,18,20,42,43,44,45,46,47,48^ A meta-analysis of 32 studies among 265 760 women reported associations of underweight, overweight, and obesity with increased risk of miscarriage.^20^ We observed that overweight, but not obesity, increased the probability of miscarriage per week of gestation compared with normal weight. This finding might be explained by the lower number of women with obesity. We observed that overweight and obesity in women were associated with increased odds of miscarriage, even when excluding couples undergoing assisted reproductive technology. Contrary to our findings, some studies did not observe associations of higher BMI in women with increased risk of miscarriage.^49,50^ When we restricted the analyses to only prepregnancy recorded weight, we observed largely similar results. We did not observe associations of BMI in men with increased odds of miscarriage. Previous studies describing the association of BMI in men and miscarriage are conflicting and were conducted mostly in populations experiencing recurrent pregnancy loss or undergoing assisted reproductive technology.^24^ Obesity in men might affect sperm quality by causing DNA damage, which could lead to embryonic chromosomal anomalies and thus negative IVF outcomes, such as miscarriage.^8,23,24,51,52^ Evidence linking male BMI with miscarriage proposes a possible association of metabolic syndrome, including obesity, with an increased risk of miscarriage.^22,53^ Further studies in the general population are needed to assess whether metabolic markers such as glucose, insulin, and cholesterol concentrations are independent of BMI associated with miscarriage risk. Thus, from the results from our study and previous studies, higher BMI in women seems to be associated with increased odds of miscarriage. The associations of BMI in men with the odds of miscarriage should be further studied.

The mechanisms underlying associations of BMI with fertility and early-pregnancy outcomes are not fully known but might include hormonal dysfunction, decreased quality of oocytes and the uterine environment, and decreased sperm quality.^41,54^ Elevated cytokine levels associated with the inflammatory state of adiposity may lead to dysregulated sex hormone secretion and metabolism, which might be adversely associated with oocyte quality and embryo implantation.^55,56,57^ A prospective cohort study among 211 subfertile men in the Netherlands reported an association of higher preconception BMI with reduced fertilization rates.^58^ Furthermore, enlarged adipose tissue acts as an endocrine organ secreting its own bioactive signaling products, such as leptin and chemerin.^59^ These products contribute to insulin resistance and dyslipidaemia, resulting in excessive androgen secretion and impaired reproductive hormone production. An excess or lack of adipose tissue, present in overweight and underweight individuals, may cause hormonal disturbances by altering the hypothalamic-pituitary-ovarian axis, leading to menstrual irregularities and anovulation.^59,60^ These mechanisms may ultimately lead to decreased fertility.

Our results suggest that preconception BMI across the full range in women and men in the general population is an important risk factor for decreased fertility and adverse (early) pregnancy outcomes. Up to 10% of women of reproductive age have underweight, and up to 40% have overweight and obesity.^55,61^ Previous studies suggest that lifestyle interventions in couples undergoing assisted reproductive technology could increase pregnancy rates, live births, and natural conceptions and could decrease time to pregnancy, suggesting that weight management before pregnancy is positively associated with fertility.^62,63,64^ Optimizing BMI in women and men in preconception care settings may be an important population health strategy to improve fertility, reduce health care costs, and prevent miscarriage.

### Strengths and Limitations

Although the strengths of this study were the prospective study design, large sample size, use of weight measurements, and inclusion of men, some limitations should be discussed. Nonresponse analyses showed that the included and excluded study populations differed, which could affect the generalizability of the results. Compared with included participants, those excluded were younger and had a higher BMI. If missing complete information on these women and men had any association with our results, we would expect that it might have led to an underestimation of the associations of BMI with time to pregnancy and miscarriage. Because not all women reported prepregnancy weight, this measurement was substituted with early-pregnancy measurements. Moreover, BMI of the included study population was not associated with age. We observed similar age distributions in participants with a BMI of less than 25 or 25 or greater. When we conducted the analyses excluding age, we observed largely similar results. Furthermore, the accuracy of time-to-pregnancy duration may have been affected by the retrospectively answered questionnaires. To address this limitation, time to pregnancy was reconfirmed during the first trimester visit. Although we adjusted for multiple confounders, residual confounding might still be an issue because of the observational nature of the study.

## Conclusions

We observed in this cohort study that BMI outside of the normal category in women and men was associated with lower fecundability, subfertility, and increased odds of miscarriage. Optimizing BMI from the preconception period onward in women and men might be an important strategy to improve fertility and pregnancy outcomes.

## Notes

Supplement 1.
eMethods.
**eFigure 1.** Flowchart of Participants Included in the Study **eFigure 2.** Directed Acyclic Graph of Body Mass Index and Time to Pregnancy **eFigure 3.** Directed Acyclic Graph of Body Mass Index and the Odds of Miscarriage **eTable 1.** Non-Response Analysis of Participants Included and Excluded From the Study Population **eTable 2.** Non-Response Analysis of Episodes Included and Excluded from the Study Population **eTable 3.** Associations of Body Mass index in Women and Men With Fecundability Ratios, Basic Model **eTable 4.** Associations of Body Mass Index in Women and Men With Fecundability Ratios, Adjusted Model **eTable 5.** Associations of Body Mass Index in Women and Men With Fecundability Ratios, Adjusted Model Excluding Top 5% of Time to Pregnancy **eTable 6.** Associations of Body Mass Index in Women and Men With Fecundability Ratios, Adjusted Model Including Only First Episodes **eTable 7.** Associations of Body Mass Index in Women and Men With Fecundability Ratios, Adjusted Model Including Body Mass Index of Partner **eTable 8.** Associations of Joint Body Mass Index of Women and Men With Fecundability Ratios, Adjusted Model **eTable 9.** Associations of Body Mass Index in Women and Men With Odds of Subfertility, Basic Model **eTable 10.** Associations of Body Mass Index in Women and Men With Odds of Subfertility, Adjusted Model **eTable 11.** Associations of Body Mass Index in Women and Men With Odds of Subfertility, Adjusted Model Excluding Couples Undergoing Assisted Reproductive Technology **eTable 12.** Associations of Body Mass Index in Women and Men With Odds of Subfertility, Adjusted Model Excluding Top 5% of Time to Pregnancy **eTable 13.** Associations of Body Mass Index in Women and Men With Odds of Subfertility, Adjusted Model Including Only First Episodes **eTable 14.** Associations of Body Mass Index in Women and Men With Odds of Subfertility, Adjusted Model Including Body Mass Index of Partner **eTable 15.** Associations of Joint Body Mass Index of Women and Men With Odds of Subfertility, Adjusted Model **eTable 16.** Associations of Body Mass Index in Women and Men With Hazard Ratios of Miscarriage, Basic Model **eTable 17.** Associations of Body Mass Index in Women and Men With Hazard Ratios of Miscarriage, Adjusted Model **eTable 18.** Associations of Body Mass Index in Women and Men With Hazard Ratios of Miscarriage, Adjusted Model Excluding Couples Undergoing Assisted Reproductive Technology **eTable 19.** Associations of Body Mass Index in Women and Men With Hazard Ratios of Miscarriage, Adjusted Model Including Only First Episodes **eTable 20.** Associations of Body Mass Index in Women and Men With Hazard Ratios of Miscarriage, Adjusted Model Including Body Mass Index of Partner **eTable 21.** Associations of Joint Body Mass Index of Women and Men With Hazard Ratios of Miscarriage, Adjusted Model **eTable 22.** Associations of Body Mass Index in Women and Men With Odds of Miscarriage, Basic Model **eTable 23.** Associations of Body Mass Index in Women and Men With Odds of Miscarriage, Adjusted Model **eTable 24.** Associations of Body Mass Index in Women and Men With Odds of Miscarriage, Adjusted Model Excluding Couples Undergoing Assisted Reproductive Technology **eTable 25.** Associations of Body Mass Index in Women and Men With Odds of Miscarriage, Adjusted Model Including Only First Episodes **eTable 26.** Associations of Body Mass Index in Women and Men With Odds of Miscarriage, Adjusted Model Including Body Mass Index of Partner **eTable 27.** Associations of Joint Body Mass Index of Women and Men With Odds of Miscarriage, Adjusted Model

Supplement 2.
Data Sharing Statement
