# Supplementary material for: Preconception and Early-Pregnancy Body Mass Index in Women and Men, Time to Pregnancy, and Risk of Miscarriage
Source: JAMA Netw Open. 2024 Sep 19;7(9):e2436157. doi: 10.1001/jamanetworkopen.2024.36157 (PMC11413718; doi:10.1001/jamanetworkopen.2024.36157)
Supplement: Supplement 1. — eMethods. eFigure 1. Flowchart of Participants Included in the Study eFigure 2. Directed Acyclic Graph of Body Mass Index and Time to Pregnancy eFigure 3. Directed Acyclic Graph of Body Mass Index and the Odds of Miscarriage eTable 1. Non-Response Analysis of Participants Included and Excluded From the Study Population eTable 2. Non-Response Analysis of Episodes Included and Excluded from the Study Population eTable 3. Associations of Body Mass index in Women and Men With Fecundability Ratios, Basic Model eTable 4. Associations of Body Mass Index in Women and Men With Fecundability Ratios, Adjusted Model eTable 5. Associations of Body Mass Index in Women and Men With Fecundability Ratios, Adjusted Model Excluding Top 5% of Time to Pregnancy eTable 6. Associations of Body Mass Index in Women and Men With Fecundability Ratios, Adjusted Model Including Only First Episodes eTable 7. Associations of Body Mass Index in Women and Men With Fecundability Ratios, Adjusted Model Including Body Mass Index of Partner eTable 8. Associations of Joint Body Mass Index of Women and Men With Fecundability Ratios, Adjusted Model eTable 9. Associations of Body Mass Index in Women and Men With Odds of Subfertility, Basic Model eTable 10. Associations of Body Mass Index in Women and Men With Odds of Subfertility, Adjusted Model eTable 11. Associations of Body Mass Index in Women and Men With Odds of Subfertility, Adjusted Model Excluding Couples Undergoing Assisted Reproductive Technology eTable 12. Associations of Body Mass Index in Women and Men With Odds of Subfertility, Adjusted Model Excluding Top 5% of Time to Pregnancy eTable 13. Associations of Body Mass Index in Women and Men With Odds of Subfertility, Adjusted Model Including Only First Episodes eTable 14. Associations of Body Mass Index in Women and Men With Odds of Subfertility, Adjusted Model Including Body Mass Index of Partner eTable 15. Associations of Joint Body Mass Index of Women and Men With Odds of Subfertility, Adjus [file jamanetwopen-e2436157-s001.pdf]

Supplementary Online Content

Boxem AJ, Blaauwendraad SM, Mulders AGMGJ, et al. Preconception and early-pregnancy body mass index in women and men, time to pregnancy, and risk of miscarriage. *JAMA Netw Open.* 2024;7(9):e2436157. doi:10.1001/jamanetworkopen.2024.36157

eMethods.
eFigure 1. Flowchart of Participants Included in the Study
eFigure 2. Directed Acyclic Graph of Body Mass Index and Time to Pregnancy
eFigure 3. Directed Acyclic Graph of Body Mass Index and the Odds of Miscarriage
eTable 1. Non-Response Analysis of Participants Included and Excluded From the Study Population
eTable 2. Non-Response Analysis of Episodes Included and Excluded from the Study Population
eTable 3. Associations of Body Mass index in Women and Men With Fecundability Ratios, Basic Model
eTable 4. Associations of Body Mass Index in Women and Men With Fecundability Ratios, Adjusted Model
eTable 5. Associations of Body Mass Index in Women and Men With Fecundability Ratios, Adjusted Model Excluding Top 5% of Time to Pregnancy
eTable 6. Associations of Body Mass Index in Women and Men With Fecundability Ratios, Adjusted Model Including Only First Episodes
eTable 7. Associations of Body Mass Index in Women and Men With Fecundability Ratios, Adjusted Model Including Body Mass Index of Partner
eTable 8. Associations of Joint Body Mass Index of Women and Men With Fecundability Ratios, Adjusted Model
eTable 9. Associations of Body Mass Index in Women and Men With Odds of Subfertility, Basic Model
eTable 10. Associations of Body Mass Index in Women and Men With Odds of Subfertility, Adjusted Model
eTable 11. Associations of Body Mass Index in Women and Men With Odds of Subfertility, Adjusted Model Excluding Couples Undergoing Assisted Reproductive Technology
eTable 12. Associations of Body Mass Index in Women and Men With Odds of Subfertility, Adjusted Model Excluding Top 5% of Time to Pregnancy
eTable 13. Associations of Body Mass Index in Women and Men With Odds of Subfertility, Adjusted Model Including Only First Episodes
eTable 14. Associations of Body Mass Index in Women and Men With Odds of Subfertility, Adjusted Model Including Body Mass Index of Partner
eTable 15. Associations of Joint Body Mass Index of Women and Men With Odds of Subfertility, Adjusted Model
eTable 16. Associations of Body Mass Index in Women and Men With Hazard Ratios of Miscarriage, Basic Model
eTable 17. Associations of Body Mass Index in Women and Men With Hazard Ratios of Miscarriage, Adjusted Model
eTable 18. Associations of Body Mass Index in Women and Men With Hazard Ratios of Miscarriage, Adjusted Model Excluding Couples Undergoing Assisted Reproductive Technology
eTable 19. Associations of Body Mass Index in Women and Men With Hazard Ratios of Miscarriage, Adjusted Model Including Only First Episodes
eTable 20. Associations of Body Mass Index in Women and Men With Hazard Ratios of Miscarriage, Adjusted Model Including Body Mass Index of Partner
eTable 21. Associations of Joint Body Mass Index of Women and Men With Hazard Ratios of Miscarriage, Adjusted Model
eTable 22. Associations of Body Mass Index in Women and Men With Odds of Miscarriage, Basic Model
eTable 23. Associations of Body Mass Index in Women and Men With Odds of Miscarriage, Adjusted Model
eTable 24. Associations of Body Mass Index in Women and Men With Odds of Miscarriage, Adjusted Model Excluding Couples Undergoing Assisted Reproductive Technology
eTable 25. Associations of Body Mass Index in Women and Men With Odds of Miscarriage, Adjusted Model Including Only First Episodes
eTable 26. Associations of Body Mass Index in Women and Men With Odds of Miscarriage, Adjusted Model Including Body Mass Index of Partner
eTable 27. Associations of Joint Body Mass Index of Women and Men With Odds of Miscarriage, Adjusted Model

This supplementary material has been provided by the authors to give readers additional information about their work.

© 2024 Boxem AJ et al. *JAMA Network Open.*

eMethods

Calculation of Fecundability Ratio on the continuous and categorical scale

The Hazard Ratio (HR) of body mass index (BMI) was calculated by dividing the hazard rate of conceiving within one month with one unit increase or decrease in BMI by the hazard rate of the mean BMI:

HR = (H(t) ± 1 unit BMI) / (H(t) mean BMI),

or, dividing the hazard rate of conceiving within one month of the different categories of BMI by the hazard rate of the reference category of BMI:

HR = (H(t) ± BMI category) / (H(t) BMI reference category).

A HR < 1 indicates a lower fecundability per unit increase or decrease in BMI or as compared to the reference category of BMI.

© 2024 Boxem AJ et al. *JAMA Network Open.*

eFigure 1. Flowchart of Participants Included in the Study

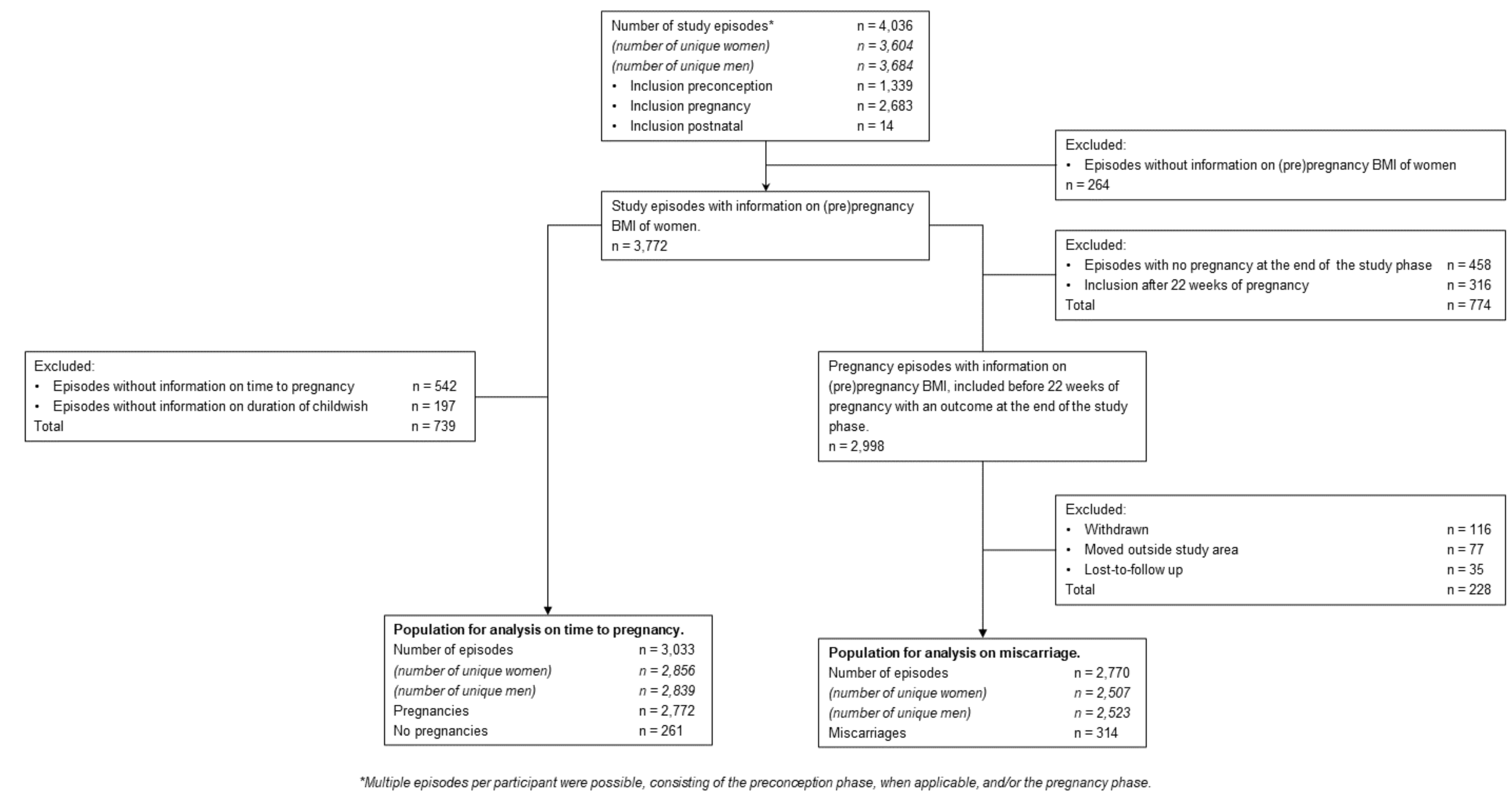

© 2024 Boxem AJ et al. *JAMA Network Open.*

eFigure 2. Directed Acyclic Graph of Body Mass Index and Time to Pregnancy

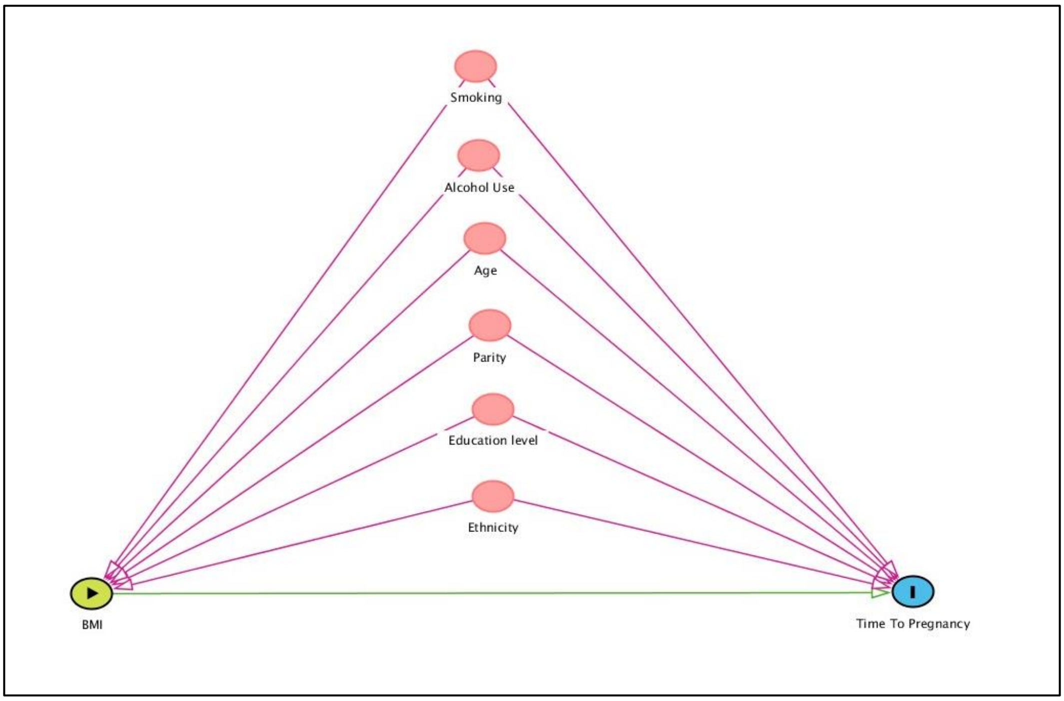

© 2024 Boxem AJ et al. *JAMA Network Open.*

eFigure 3. Directed Acyclic Graph of Body Mass Index and the Odds of Miscarriage

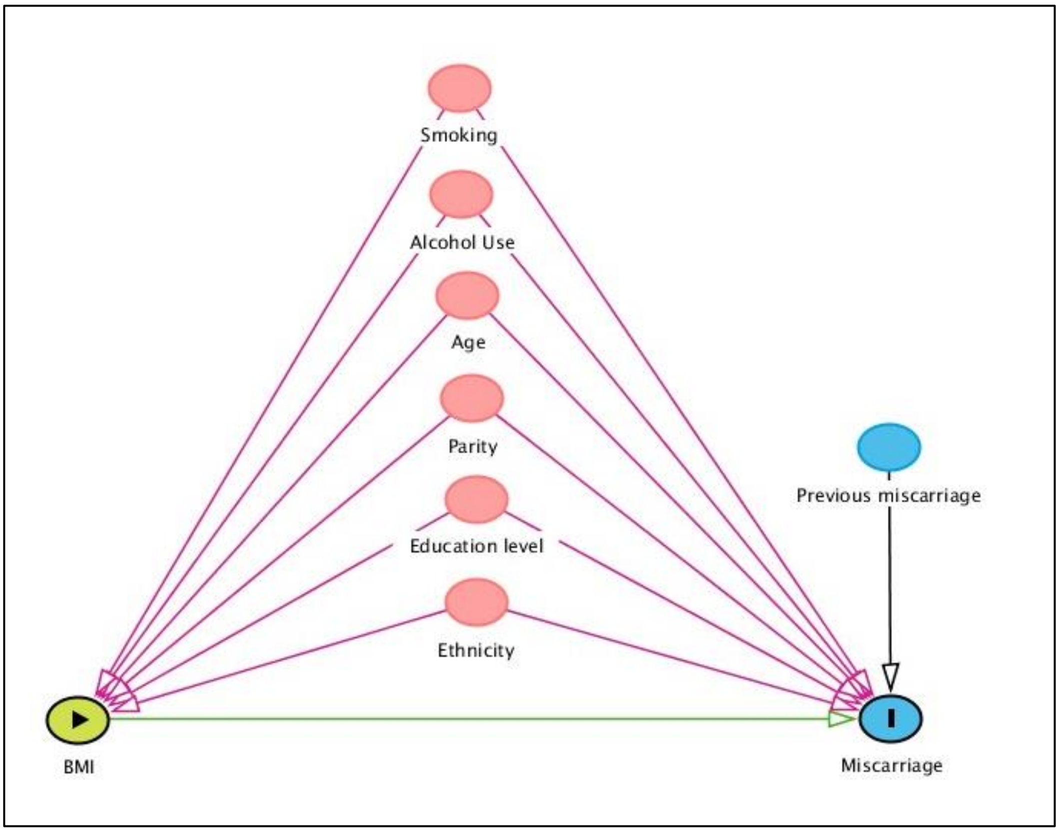

© 2024 Boxem AJ et al. *JAMA Network Open.*

eTable 1. Non-Response Analysis of Participants Included and Excluded From the Study Population

	Women			Men		
	**Participants, No. %**			**Participants, No. %**		
	Responders	Non-responders	p-value	Responders	Non-responders	p-value
	N=3,160[a]	N=238[b]		N=2,272[a]	N=39[b]	
Age at enrolment, median (IQR), y	31.3 (28.8-34.3)	30.0 (27.1-33.6)	*<0.01*	33.3 (30.3-36.6)	29.9 (26.5-34.7)	*<0.01*
<30 y	1126 (35.6)	118 (49.6)		530 (23.3)	21 (53.8)	
30-35 y	1369 (43.3)	77 (32.4)		933 (41.1)	9 (23.1)	
>35 y	665 (21.0)	43 (18.1)		807 (35.6)	9 (23.1)	
Missing	0 (0.0)	0 (0.0)		2 (0.1)	0 (0.0)	
Ethnicity			*<0.01*			*0.12*
Dutch	1775 (60.0)	64 (48.5)		1409 (65.3)	8 (47.1)	
Other non-Western [c]	275 (9.3)	21 (15.9)		201 (9.3)	1 (5.9)	
Other Western [d]	906 (30.6)	47 (35.6)		548 (25.4)	8 (47.1)	
Missing	204 (6.5)	106 (44.5)		114 (5.0)	22 (56.4)	
Educational level			*0.85*			*0.07*
No, primary or secondary education finished	889 (30.3)	38 (29.2)		777 (36.0)	10 (58.8)	
Higher education finished	2046 (69.7)	92 (70.8)		1382 (64.0)	7 (41.2)	
Missing	225 (7.1)	108 (45.4)		113 (5.0)	22 (56.4)	
BMI, median (IQR)	23.6 (21.3-26.9)	24.1 (21.8-28.4)	*0.02*	25.0 (23.0-27.5)	26.8 (23.4-28.9)	*0.08*
Underweight (<18.5)	96 (3.0)	4 (1.7)		19 (0.8)	0 (0.0)	
Normal weight (18.5-24.9)	1879 (59.5)	128 (53.8)		1124 (49.5)	14 (35.9)	
Overweight (25-29.9)	752 (23.8)	57 (23.9)		862 (37.9)	19 (48.7)	
Obesity (≥30)	433 (13.7)	49 (20.6)		267 (11.8)	6 (15.4)	
Missing	0 (0.0)	0 (0.0)		0 (0.0)	0 (0.0)	
Smoking			*0.02*			*0.55*
No	1580 (55.3)	65 (55.6)		1060 (50.2)	7 (50.0)	
Quit smoking before pregnancy	875 (30.6)	45 (38.5)		660 (31.3)	3 (21.4)	
Smoked during pregnancy	400 (14.0)	7 (6.0)		390 (18.5)	4 (28.6)	
Missing	305 (9.7)	121 (50.8)		162 (7.1)	25 (64.1)	

© 2024 Boxem AJ et al. *JAMA Network Open.*

	Women			Men		
	Participants, No. %			Participants, No. %		
	Responders	Non-responders	p-value	Responders	Non-responders	p-value
	N=3,160[a]	N=238[b]		N=2,272[a]	N=39[b]	
Alcohol consumption			*<0.01*			*0.19*
No consumption < 3 mo before pregnancy	638 (22.3)	21 (18.3)		255 (12.1)	4 (26.7)	
Consumption < 3 mo before pregnancy	1803 (63.1)	88 (76.5)		1850 (87.9)	11 (73.3)	
Consumption during pregnancy	418 (14.6)	6 (5.2)		NA	NA	
Missing	301 (9.5)	123 (51.7)		176 (7.4)	24 (61.5)	
Parity			*<0.01*			*1.00*
Nulliparous	1933 (67.8)	98 (83.1)		1479 (69.7)[f]	8 (72.7)[f]	
Multiparous	918 (32.2)	20 (16.9)		643 (30.3)[f]	3 (27.3)[f]	
Missing	309 (9.8)	120 (50.4)		150 (6.6)[f]	28 (71.8)[f]	
Miscarriage in previous pregnancy			*0.04*			*0.64*
No	2293 (80.9)	105 (89.0)		1740 (82.3)[f]	11 (91.7)[f]	
Yes	543 (19.1)	13 (11.0)		375 (17.7)[f]	1 (8.3)[f]	
Missing	324 (10.3)	120 (50.4)		157 (6.9)[f]	28 (71.8)[f]	
Time to pregnancy, median (95% range), mo.[e]	3.4 (0.0-67.9)	NA	*NA*	3.0 (0.0-58.7)[f]	NA[f]	*NA*
0-12 mo	1825 (65.2)	0		1582 (75.9)[f]	0[f]	
> 12 mo	463 (16.5)	0		311 (14.9)[f]	0[f]	
ART leading to pregnancy	264 (9.4)	0		192 (9.2)[f]	0[f]	
Not pregnant	249 (8.9)	155 (100.0)		0[f]	0[f]	
Missing	359 (11.4)	83 (34.9)		187 (8.2)[f]	39 (100.0)[f]	
Occurrence of miscarriage			*NA*			*NA*
Miscarriage	280 (9.6)	0		151 (6.6)[f]	0[f]	
No miscarriage	2631 (90.4)	83 (100.0)		2121 (93.4)[f]	39 (100.0)[f]	
Missing	249 (7.9)	155 (65.1)		0[f]	0[f]	
Timing of miscarriage, median (IQR), wk.	8.3 (7.1-9.6)	NA	*NA*	8.7 (7.9-9.6)[f]	NA[f]	*NA*
First trimester	255 (92.7)	0		138 (92.0)[f]	0[f]	
Second trimester	20 (7.3)	0		12 (8.0)[f]	0[f]	
Missing	5 (1.8)	238 (100.0)		1 (0.7)[f]	39 (100.0)[f]	

© 2024 Boxem AJ et al. *JAMA Network Open.*

Abbreviations: ART, assisted reproductive technology; BMI, body mass index (calculated as weight in kilograms divided by height in meters squared); NA, not applicable.
Women were included in preconception and pregnancy between 2017 and 2021. Values are presented as median (IQR), median (95% range) or number of participants (valid %).
[a] Study population of time to pregnancy and miscarriage consisting of 3,160 unique women and 2,272 unique men from Rotterdam.
[b] Non-responders consisted of 238 unique women and 39 unique men.
[c] Included: African; American, non-western; Asian, non-western; Chinese; Indonesian ethnicity.
[d] Included: American, western; Asian, western; Cape Verdean; Dutch Antilles; European; German; Yugoslav; Moroccan; Oceanian; Polish; Surinamese, or Turkish.
[e] Time to pregnancy in months was derived from pregnancy episodes with a natural conception.
[f] Parity, miscarriage in previous pregnancy, time to pregnancy in months, occurrence of miscarriage, timing of miscarriage in weeks in men were derived from their partner.

© 2024 Boxem AJ et al. *JAMA Network Open.*

eTable 2. Non-Response Analysis of Episodes Included and Excluded from the Study Population

	Women			Men		
	Episodes, No. %			Episodes, No. %		
	Responders	Non-responders	p-value	Responders	Non-responders	p-value
	N = 3,476[a]	N = 296[b]		N = 2,513[a]	N = 49[b]	
Age at enrolment, median (IQR), y	31.5 (28.9-34.5)	30.6 (27.4-34.1)	*0.01*	33.4 (30.4-36.8)	29.9 (26.4-35.1)	*<0.01*
<30 y	1193 (34.3)	135 (45.6)		565 (22.5)	26 (53.1)	
30-35 y	1527 (43.9)	101 (34.1)		1017 (40.5)	10 (20.4)	
>35 y	756 (21.7)	60 (20.3)		929 (37.0)	13 (26.5)	
Missing	0	0		2 (0.1)	0	
Ethnicity			*0.01*			*0.03*
Dutch	1987 (60.9)	91 (50.0)		1569 (65.5)	12 (48.0)	
Other non-Western[c]	295 (9.0)	23 (12.6)		222 (9.3)	1 (4.0)	
Other Western[d]	983 (30.1)	68 (37.4)		606 (25.3)	12 (48.0)	
Missing	211 (6.1)	114 (38.5)		116 (4.6)	24 (49.0)	
Educational level			*0.93*			*0.04*
No, primary or secondary education finished	961 (29.6)	54 (29.8)		843 (35.2)	14 (56.0)	
Higher education finished	2283 (70.4)	127 (70.2)		1555 (64.8)	11 (44.0)	
Missing	232 (6.7)	115 (38.9)		115 (4.6)	24 (49.0)	
BMI, median (IQR)	23.6 (21.3-26.9)	24.1 (21.6-28.3)	*0.01*	25.0 (23.0-27.5)	26.1 (22.6-28.7)	*0.32*
Underweight (<18.)	103 (3.0)	6 (2.0)		20 (0.8)	1 (2.0)	
Normal weight (18.5-24.9)	2067 (59.5)	158 (53.4)		1236 (49.2)	20 (40.8)	
Overweight (25-29.9)	835 (24.0)	72 (24.3)		962 (38.3)	20 (40.8)	
Obesity (≥30)	471 (13.6)	60 (20.3)		295 (11.7)	8 (16.3)	
Missing	0	0		0	0	
Smoking			*0.08*			*0.46*
No	1612 (54.9)	65 (54.6)		1181 (50.9)	7 (41.2)	
Quit smoking before pregnancy	913 (31.1)	45 (37.8)		725 (31.2)	5 (29.4)	
Smoked during pregnancy	413 (14.1)	9 (7.6)		416 (17.9)	5 (29.4)	
Missing	538 (15.5)	177 (59.8)		191 (7.6)	32 (65.3)	

© 2024 Boxem AJ et al. *JAMA Network Open.*

	Women			Men		
	Episodes, No. %			Episodes, No. %		
	Responders	Non-responders	p-value	Responders	Non-responders	p-value
	N = 3,476[a]	N = 296[b]		N = 2,513[a]	N = 49[b]	
Alcohol consumption			*<0.01*			*0.09*
No consumption < 3 mo before pregnancy	686 (22.0)	21 (17.2)		276 (11.9)	5 (27.8)	
Consumption < 3 mo before pregnancy	1972 (63.3)	93 (76.2)		2042 (88.1)	13 (72.2)	
Consumption during pregnancy	458 (14.7)	8 (6.6)		NA	NA	
Missing	360 (10.4)	174 (58.8)		195 (7.8)	31 (63.3)	
Parity			*0.30*			*0.07*
Nulliparous	2042 (64.8)	116 (69.0)		1567 (66.5)[f]	9 (45.0)[f]	
Multiparous	1108 (35.2)	52 (31.0)		791 (33.5)[f]	11 (55.0)[f]	
Missing	326 (9.4)	128 (43.2)		155 (6.2)[f]	29 (59.2)[f]	
Miscarriage in previous pregnancy			*0.01*			*0.56*
No	2302 (79.3)	105 (89.0)		1743 (80.8)[f]	11 (91.7)[f]	
Yes	601 (20.7)	13 (11.0)		415 (19.2)[f]	1 (8.3)[f]	
Missing	573 (16.5)	178 (60.1)		355 (14.1)[f]	37 (75.5)[f]	
Time to pregnancy, median (95% range), mo.[e]	3.7 (0.0-68.3)	NA	*NA*	3.3 (0.0-59.5)[f]	NA[f]	*NA*
0-12 mo	1944 (63.7)	0		1692 (74.0)[f]	0[f]	
> 12 mo	541 (17.7)	0		382 (16.7)[f]	0[f]	
ART leading to pregnancy	306 (10.0)	0		214 (9.4)[f]	0[f]	
Not pregnant	261 (8.6)	197 (100.0)		0[f]	0[f]	
Missing	424 (12.2)	99 (33.4)		225 (9.0)[f]	49 (100.0)[f]	
Occurrence of miscarriage			*NA*			*NA*
Miscarriage	314 (9.8)	0		174 (6.9)[f]	0[f]	
No miscarriage	2901 (90.2)	21 (100.0)		2339 (93.1)[f]	49 (100.0)[f]	
Missing	261 (7.5)	275 (92.3)		0[f]	0[f]	
Timing of miscarriage, median (IQR), wk.	8.3 (0.8-53.8)	NA	*NA*	8.6 (7.9-9.6)[f]	NA[f]	*NA*
First trimester	284 (90.2)	0		158 (91.9)[f]	0[f]	
Second trimester	24 (7.6)	0		14 (8.1)[f]	0[f]	
Missing	7 (2.2)	296 (100.0)		2 (1.1)[f]	49 (100.0)[f]	

© 2024 Boxem AJ et al. *JAMA Network Open.*

Abbreviations: ART, assisted reproductive technology; BMI, body mass index (calculated as weight in kilograms divided by height in meters squared); NA, not applicable.
Women were included in preconception and pregnancy between 2017 and 2021. Values are presented as median (IQR), median (95% range) or number of participants (valid %).
[a] Study population of time to pregnancy and miscarriage consisting of 3,183 unique women and 2,330 unique men from Rotterdam, the Netherlands with a total of 3,476 and 2,513 study episodes.
[b] Non-responders consisted of 296 unique women and 49 unique men with a total of 296 and 49 study episodes.
[c] Included: African; American, non-western; Asian, non-western; Chinese; Indonesian ethnicity.
[d] Included: American, western; Asian, western; Cape Verdean; Dutch Antilles; European; German; Yugoslav; Moroccan; Oceanian; Polish; Surinamese, or Turkish.
[e] Time to pregnancy in months was derived from pregnancy episodes with a natural conception.
[f] Parity, miscarriage in previous pregnancy, time to pregnancy in months, occurrence of miscarriage, timing of miscarriage in weeks in men were derived from their partner.

© 2024 Boxem AJ et al. *JAMA Network Open.*

eTable 3. Associations of Body Mass index in Women and Men With Fecundability Ratios, Basic Model

	Women		Men	
	N	FR (95% CI)	N	FR (95% CI)
Body mass index in kg/m^2	2,739	0.97 (0.96-0.97)	2,074	0.98 (0.96-0.99)
Underweight (<18.5)	83	0.81 (0.64-1.02)	15	1.14 (0.68-1.89)
Normal weight (18.5-24.9)	1,699	Reference	1,049	Reference
Overweight (25.0-29.9)	626	0.81 (0.74-0.89)	796	0.88 (0.80-0.96)
Obesity (≥30.0)	331	0.61 (0.54-0.69)	214	0.76 (0.65-0.88)

Values represent the fecundability per unit increase in and categories of body mass index (95% confidence interval (CI)), as compared to the reference category. Fecundability represents the probability of conceiving within one month. Models were analysed using Cox proportional hazards models . Fecundability ratios were derived from the Hazard Ratios of the Cox proportional hazards models.

© 2024 Boxem AJ et al. *JAMA Network Open.*

eTable 4. Associations of Body Mass Index in Women and Men With Fecundability Ratios, Adjusted Model

	Women		Men	
	N	FR (95% CI)	N	FR (95% CI)
Body mass index in kg/m^2	2,739	0.98 (0.97-0.99)	2,074	0.99 (0.98-1.00)
Underweight (<18.5)	83	0.84 (0.67-1.06)	15	1.21 (0.72-2.03)
Normal weight (18.5-24.9)	1,699	Reference	1,049	Reference
Overweight (25.0-29.9)	626	0.88 (0.80-0.98)	796	0.94 (0.85-1.03)
Obesity (≥30.0)	331	0.72 (0.63-0.82)	214	0.87 (0.74-1.01)

Values represent the fecundability per unit increase in and categories of body mass index (95% confidence interval (CI)), as compared to the reference category. Fecundability represents the probability of conceiving within one month. Models were analysed using Cox proportional hazards models. Fecundability ratios were derived from the Hazard Ratios of the Cox proportional hazards models. Models were adjusted for participants age, ethnicity, education level, smoking and alcohol consumption. Parity was included in the women’s models.

© 2024 Boxem AJ et al. *JAMA Network Open.*

eTable 5. Associations of Body Mass Index in Women and Men With Fecundability Ratios, Adjusted Model Excluding Top 5% of Time to Pregnancy

	**Women**		**Men**	
	N	FR (95% CI)	N	FR (95% CI)
Body mass index in kg/m^2	2,602	0.98 (0.97-0.99)	2,015	0.99 (0.98-1.00)
Underweight (<18.5)	78	0.87 (0.68-1.10)	15	1.06 (0.63-1.77)
Normal weight (18.5-24.9)	1,639	Reference	1,028	Reference
Overweight (25.0-29.9)	591	0.87 (0.79-0.97)	769	0.93 (0.84-1.03)
Obesity (≥30.0)	294	0.80 (0.69-0.92)	203	0.87 (0.74-1.02)

Values represent the fecundability per unit increase in and categories of body mass index (95% confidence interval (CI)), as compared to the reference category. Fecundability represents the probability of conceiving within one month. Models were analysed using Cox proportional hazards models. Fecundability ratios were derived from the Hazard Ratios of the Cox proportional hazards models. Models were adjusted for participants age, ethnicity, education level, smoking and alcohol consumption. Parity was included in the women's models. The top 5% of time to pregnancy observations were excluded.

© 2024 Boxem AJ et al. *JAMA Network Open.*

eTable 6. Associations of Body Mass Index in Women and Men With Fecundability Ratios, Adjusted Model Including Only First Episodes

	**Women**		**Men**	
	N	FR (95% CI)	N	FR (95% CI)
Body mass index in kg/m^2	2,530	0.98 (0.97-0.99)	1,893	0.99 (0.98-1.00)
Underweight (<18.5)	80	0.82 (0.65-1.04)	14	1.44 (0.85-2.46)
Normal weight (18.5-24.9)	1,576	Reference	966	Reference
Overweight (25.0-29.9)	565	0.89 (0.80-0.99)	719	0.95 (0.86-1.05)
Obesity (≥30.0)	309	0.73 (0.63-0.84)	194	0.86 (0.73-1.01)

Values represent the fecundability per unit increase in and categories of body mass index (95% confidence interval (CI)), as compared to the reference category Fecundability represents the probability of conceiving within one month. Models were analysed using Cox proportional hazards models. Fecundability ratios were derived from the Hazard Ratios of the Cox proportional hazards models. Models were adjusted for participants age, ethnicity, education level, smoking and alcohol consumption. Parity was included in the women's model. Only the first episodes were included in the model.

© 2024 Boxem AJ et al. *JAMA Network Open.*

eTable 7. Associations of Body Mass Index in Women and Men With Fecundability Ratios, Adjusted Model Including Body Mass Index of Partner

	**Women**		**Men**	
	N	FR (95% CI)	N	FR (95% CI)
Body mass index in kg/m^2	2,074	1.00 (0.99-1.01)	2,074	0.99 (0.98-1.00)
Underweight (<18.5)	62	0.93 (0.72-1.20)	15	1.22 (0.73-2.04)
Normal weight (18.5-24.9)	1,367	Reference	1,049	Reference
Overweight (25.0-29.9)	436	0.99 (0.88-1.10)	796	0.94 (0.86-1.03)
Obesity (≥30.0)	209	0.93 (0.80-1.09)	214	0.89 (0.76-1.04)

Values represent the fecundability per unit increase in and categories of body mass index (95% confidence interval (CI)), as compared to the reference category. Fecundability represents the probability of conceiving within one month. Models were analysed using Cox proportional hazards models. Fecundability ratios were derived from the Hazard Ratios of the Cox proportional hazards models. Models were adjusted for body mass index of partner, participants age, ethnicity, education level, smoking and alcohol consumption. Parity was included in the women’s model.

© 2024 Boxem AJ et al. *JAMA Network Open.*

eTable 8. Associations of Joint Body Mass Index of Women and Men With Fecundability Ratios, Adjusted Model

	Women and men combined	
	Effect Estimate	p-value
Interaction term body mass index women * body mass index men	1.00	0.71
	N	FR (95% CI)
Normal weight in both women and men (18.5-24.9 kg/m^2)	791	Reference
Normal weight in women (18.5-24.9 kg/m2) * overweight and obesity in men (≥25.0 kg/m^2)	569	0.89 (0.80-1.00)
Overweight and obesity in women (≥25.0 kg/m2) * normal weight in men (18.5-24.9 kg/m^2)	222	0.95 (0.82-1.11)
Overweight and obesity in both women and men (≥25.0 kg/m^2)	415	0.92 (0.80-1.04)

Values represent the fecundability per categories of body mass index (95% confidence interval (CI)), as compared to the reference category Fecundability represents the probability of conceiving within one month. Models were analysed using Cox proportional hazards models. Fecundability ratios were derived from the Hazard Ratios of the Cox proportional hazards models. Models were adjusted for age, ethnicity, education level, smoking and alcohol consumption of both women and men, and parity.

© 2024 Boxem AJ et al. *JAMA Network Open.*

eTable 9. Associations of Body Mass Index in Women and Men With Odds of Subfertility, Basic Model

	Women		Men	
	N	OR (95% CI)	N	OR (95% CI)
Body mass index in kg/m^2	3,018	1.05 (1.04-1.07)	2,294	1.05 (1.02-1.08)
Underweight (<18.5)	97	1.87 (1.24-2.82)	17	1.02 (0.33-3.17)
Normal weight (18.5-24.9)	1,846	Reference	1,143	Reference
Overweight (25.0-29.9)	710	1.52 (1.27-1.82)	884	1.25 (1.02-1.53)
Obesity (≥30.0)	365	2.10 (1.67-2.63)	250	1.97 (1.47-2.64)

Values represent the odds of subfertility (time to pregnancy > 12 months) per unit increase in and categories of body mass index (95% confidence interval (CI)) from logistic regression models, as compared to the reference category.

© 2024 Boxem AJ et al. *JAMA Network Open.*

eTable 10. Associations of Body Mass Index in Women and Men With Odds of Subfertility, Adjusted Model

	**Women**		**Men**	
	N	OR (95% CI)	N	OR (95% CI)
Body mass index in kg/m^2	3,018	1.04 (1.02-1.05)	2,294	1.03 (1.00-1.06)
Underweight (<18.5)	97	1.88 (1.22-2.88)	17	1.11 (0.35-3.48)
Normal weight (18.5-24.9)	1,846	Reference	1,143	Reference
Overweight (25.0-29.9)	710	1.35 (1.11-1.63)	884	1.13 (0.92-1.39)
Obesity (≥30.0)	365	1.67 (1.30-2.13)	250	1.69 (1.24-2.31)

Values represent the odds of subfertility (time to pregnancy > 12 months) per unit increase in and categories of body mass index (95% confidence interval (CI)) from logistic regression models, as compared to the reference category. Models were adjusted for participants age, ethnicity, education level, smoking and alcohol consumption. Parity was included in the women's model.

© 2024 Boxem AJ et al. *JAMA Network Open.*

eTable 11. Associations of Body Mass Index in Women and Men With Odds of Subfertility, Adjusted Model Excluding Couples Undergoing Assisted Reproductive Technology

	**Women**		**Men**	
	N	OR (95% CI)	N	OR (95% CI)
Body mass index in kg/m^2	2,703	1.04 (1.02-1.06)	2,074	1.02 (0.99-1.05)
Underweight (<18.5)	82	1.71 (1.06-2.78)	15	0.75 (0.17- 3.41)
Normal weight (18.5-24.9)	1,676	Reference	1,049	Reference
Overweight (25.0-29.9)	620	1.25 (1.01-1.55)	796	1.08 (0.84-1.38)
Obesity (≥30.0)	325	1.69 (1.29-2.21)	214	1.45 (1.00-2.09)

Values represent the odds of subfertility (time to pregnancy > 12 months) per unit increase in and categories of body mass index (95% confidence interval (CI)) from logistic regression models, as compared to the reference category. Models were adjusted for participants age, ethnicity, education level, smoking, alcohol consumption, and parity in the women's model, and stratified for assisted reproductive technology.

© 2024 Boxem AJ et al. *JAMA Network Open.*

eTable 12. Associations of Body Mass Index in Women and Men With Odds of Subfertility, Adjusted Model Excluding Top 5% of Time to Pregnancy

	Women		Men	
	N	OR (95% CI)	N	OR (95% CI)
Body mass index in kg/m^2	2,867	1.03 (1.01-1.05)	2,222	1.03 (1.00-1.06)
Underweight (<18.5)	93	1.97 (1.28-3.04)	17	1.19 (0.38-3.71)
Normal weight (18.5-24.9)	1,773	Reference	1,118	Reference
Overweight (25.0-29.9)	669	1.36 (1.12-1.65)	851	1.10 (0.88-1.37)
Obesity (≥30.0)	332	1.64 (1.27- 2.12)	236	1.62 (1.17-2.24)

Values represent the odds of subfertility (time to pregnancy > 12 months) per unit increase in and categories of body mass index (95% confidence interval (CI)) from logistic regression models, as compared to the reference category. Models were adjusted for participants age, ethnicity, education level, smoking and alcohol consumption. Parity was included in the women's model. The top 5% of time to pregnancy observations were excluded.

© 2024 Boxem AJ et al. *JAMA Network Open.*

eTable 13. Associations of Body Mass Index in Women and Men With Odds of Subfertility, Adjusted Model Including Only First Episodes

	**Women**		**Men**	
	N	OR (95% CI)	N	OR (95% CI)
Body mass index in kg/m^2	2,775	1.04 (1.02-1.06)	2,088	1.03 (1.01-1.06)
Underweight (<18.5)	92	1.91 (1.23-2.97)	16	0.94 (0.26-3.37)
Normal weight (18.5-24.9)	1,701	Reference	1,048	Reference
Overweight (25.0-29.9)	645	1.37 (1.12-1.67)	798	1.14 (0.91-1.43)
Obesity (≥30.0)	337	1.68 (1.30-2.18)	226	1.84 (1.32-2.57)

Values represent the odds of subfertility (time to pregnancy > 12 months) per unit increase in and categories of body mass index (95% confidence interval (CI)) from logistic regression models, as compared to the reference category. Models were adjusted for participants age, ethnicity, education level, smoking and alcohol consumption. Parity was included in the women's model. Only the first episodes were included in the model.

© 2024 Boxem AJ et al. *JAMA Network Open.*

eTable 14. Associations of Body Mass Index in Women and Men With Odds of Subfertility, Adjusted Model Including Body Mass Index of Partner

	**Women**		**Men**	
	N	OR (95% CI)	N	OR (95% CI)
Body mass index in kg/m^2	2,294	1.02 (1.00-1.04)	2,294	1.02 (1.00-1.05)
Underweight (<18.5)	73	1.69 (1.00-2.86)	17	1.10 (0.35-3.46)
Normal weight (18.5-24.9)	1,491	Reference	1,143	Reference
Overweight (25.0-29.9)	498	1.22 (0.96-1.55)	884	1.10 (0.89-1.37)
Obesity (≥30.0)	232	1.32 (0.96-1.83)	250	1.58 (1.15-2.18)

Values represent the odds of subfertility (time to pregnancy > 12 months) per unit increase in and categories of body mass index (95% confidence interval (CI)) from logistic regression models, as compared to the reference category. Models were adjusted for body mass index of partner, participants age, ethnicity, education level, smoking and alcohol consumption. Parity was included in the women's model.

© 2024 Boxem AJ et al. *JAMA Network Open.*

eTable 15. Associations of Joint Body Mass Index of Women and Men With Odds of Subfertility, Adjusted Model

**Total population**		
	**Men**	
**Women**	Normal weight (18.5-24.9 kg/m^2)	Overweight and obesity (≥25.0 kg/m^2)
Normal weight (18.5-24.9 kg/m^2)	920 (59)	704 (37)
Overweight and obesity (≥25.0 kg/m^2)	288 (27)	552 (47)
* NA = 1,572 of 4,036 episodes		
**Time to pregnancy population, stratified for subfertility (>12 months)**		
	**Men**	
**Women**	Normal weight (18.5-24.9 kg/m^2)	Overweight and obesity (≥25.0 kg/m^2)
Normal weight (18.5-24.9 kg/m^2)	851 (178)	632 (169)
Overweight and obesity (≥25.0 kg/m^2)	249 (73)	473 (155)
* NA = 813 of 3,018 episodes		

	**Women and men combined**	
	Effect Estimate	p-value
Interaction term body mass index women * body mass index men	1.00	0.63
	N	OR (95% CI)
Normal weight in both women and men (18.5-24.9 kg/m^2)	851	Reference
Normal weight in women (18.5-24.9 kg/m2) * overweight and obesity in men (≥25.0 kg/m^2)	632	1.27 (0.98-1.64)
Overweight and obesity in women (≥25.0 kg/m2) * normal weight in men (18.5-24.9 kg/m^2)	249	1.35 (0.97-1.89)
Overweight and obesity in both women and men (≥25.0 kg/m^2)	473	1.41 (1.06-1.87)

Values represent the odds of subfertility (95% confidence interval (CI)) from logistic regression models, as compared to the reference category. Models were adjusted for age, ethnicity, education level, smoking and alcohol consumption of both women and men, and parity and history of miscarriage.

© 2024 Boxem AJ et al. *JAMA Network Open.*

eTable 16. Associations of Body Mass Index in Women and Men With Hazard Ratios of Miscarriage, Basic Model

	Women		Men	
	N	HR (95% CI)	N	HR (95% CI)
Body mass index in kg/m^2	2,763	1.01 (0.99-1.04)	2,186	1.01 (0.97-1.05)
Underweight (<18.5)	71	0.97 (0.46- 2.08)	16	0.00 (0.00-Inf)
Normal weight (18.5-24.9)	1,686	Reference	1,079	Reference
Overweight (25.0-29.9)	653	1.42 (1.10-1.83)	838	1.00 (0.72-1.38)
Obesity (≥30.0)	353	1.26 (0.90-1.76)	253	0.99 (0.53-1.47)

Values represent the Hazard Ratio of miscarriage per unit increase in and categories of body mass index (95% confidence interval (CI)) as compared to the reference category. Models were analysed using Cox proportional hazards models.

© 2024 Boxem AJ et al. *JAMA Network Open.*

eTable 17. Associations of Body Mass Index in Women and Men With Hazard Ratios of Miscarriage, Adjusted Model

	Women		Men	
	N	HR (95% CI)	N	HR (95% CI)
Body mass index in kg/m^2	2,763	1.02 (0.99-1.04)	2,186	0.99 (0.95-1.04)
Underweight (<18.5)	71	0.99 (0.46-2.13)	16	0.00 (0.00-Inf)
Normal weight (18.5-24.9)	1,686	Reference	1,079	Reference
Overweight (25.0-29.9)	653	1.43 (1.10-1.86)	838	0.93 (0.67-1.30)
Obesity (≥30.0)	353	1.33 (0.94-1.88)	253	0.79 (0.47-1.35)

Values represent the Hazard Ratio of miscarriage per unit increase in and categories of body mass index (95% confidence interval (CI)) as compared to the reference category). Models were analysed using Cox proportional hazards models. Models were adjusted for participants age, ethnicity, education level, smoking and alcohol consumption. Parity and history of miscarriage were included in the women's model.

© 2024 Boxem AJ et al. *JAMA Network Open.*

eTable 18. Associations of Body Mass Index in Women and Men With Hazard Ratios of Miscarriage, Adjusted Model Excluding Couples Undergoing Assisted Reproductive Technology

	**Women**		**Men**	
	N	HR (95% CI)	N	HR (95% CI)
Body mass index in kg/m^2	2,499	1.02 (0.99-1.05)	1,995	1.00 (0.96-1.04)
Underweight (<18.5)	60	1.15 (0.54-2.47)	14	0.00 (0.00-Inf)
Normal weight (18.5-24.9)	1,537	Reference	996	Reference
Overweight (25-29.9)	581	1.39 (1.05-1.85)	765	0.96 (0.68-1.35)
Obesity (≥30.0)	321	1.39 (0.97-2.00)	220	0.82 (0.47-1.44)

Values represent the Hazard Ratio of miscarriage per unit increase in and categories of body mass index (95% confidence interval (CI)) as compared to the reference category. Models were analysed using Cox proportional hazards models. Models were adjusted for participants age, ethnicity, education level, smoking, alcohol consumption, parity and history of miscarriage in the women's model, and stratified for assisted reproductive technology.

© 2024 Boxem AJ et al. *JAMA Network Open.*

eTable 19. Associations of Body Mass Index in Women and Men With Hazard Ratios of Miscarriage, Adjusted Model Including Only First Episodes

	**Women**		**Men**	
	N	HR (95% CI)	N	HR (95% CI)
Body mass index in kg/m^2	2,477	1.02 (0.99-1.04)	1,963	1.00 (0.96-1.05)
Underweight (<18.5)	65	1.07 (0.50-2.29)	16	0.00 (0.00-Inf)
Normal weight (18.5-24.9)	1,513	Reference	979	Reference
Overweight (25.0-29.9)	578	1.43 (1.08-1.89)	740	0.92 (0.65-1.32)
Obesity (≥30.0)	321	1.30 (0.90-1.88)	228	0.81 (0.46-1.42)

Values represent the Hazard Ratio of miscarriage per unit increase in and categories of body mass index (95% confidence interval (CI)) as compared to the reference category. Models were analysed using Cox proportional hazards models. Models were adjusted for participants age, ethnicity, education level, smoking and alcohol consumption. Parity and history of miscarriage were included in the women's model. Only the first episodes were included in the model.

© 2024 Boxem AJ et al. *JAMA Network Open.*

eTable 20. Associations of Body Mass Index in Women and Men With Hazard Ratios of Miscarriage, Adjusted Model Including Body Mass Index of Partner

	**Women**		**Men**	
	N	HR (95% CI)	N	HR (95% CI)
Body mass index in kg/m^2	2,186	1.02 (0.99-1.06)	2,186	0.99 (0.95-1.03)
Underweight (<18.5)	59	0.98 (0.36-2.71)	16	0.00 (000-Inf)
Normal weight (18.5-24.9)	1,417	Reference	1,079	Reference
Overweight (25.0-29.9)	471	1.68 (1.18-2.38)	838	0.91 (0.66-1.27)
Obesity (≥30.0)	239	1.33 (0.80-2.20)	253	0.74 (0.43-1.28)

Values represent the Hazard Ratio of miscarriage per unit increase in and categories of body mass index (95% confidence interval (CI)) as compared to the reference category. Models were analysed using Cox proportional hazards models. Models were adjusted for body mass index of partner, participants age, ethnicity, education level, smoking and alcohol consumption. Parity and history of miscarriage were included in the women's model.

© 2024 Boxem AJ et al. *JAMA Network Open.*

eTable 21. Associations of Joint Body Mass Index of Women and Men With Hazard Ratios of Miscarriage, Adjusted Model

	**Women and men combined**	
	Effect Estimate	p-value
Interaction term body mass index women * body mass index men	1.00	0.57
	N	HR (95% CI)
Normal weight in both women and men (18.5-24.9 kg/m^2)	799	Reference
Normal weight in women (18.5-24.9 kg/m^2) * overweight and obesity in men (≥25.0 kg/m^2)	608	0.81 (0.53-1.23)
Overweight and obesity in women (≥25.0 kg/m^2) * normal weight in men (18.5-24.9 kg/m^2)	241	1.37 (0.85-2.22)
Overweight and obesity in both women and men (≥25.0 kg/m^2)	464	1.33 (0.87-2.04)

Values represent the Hazard Ratio of miscarriage (95% confidence interval (CI)) from logistic regression models, as compared to the reference category. Models were analysed using Cox proportional hazards models. Models were adjusted for age, ethnicity, education level, smoking and alcohol consumption of both women and men, and parity and history of miscarriage.

© 2024 Boxem AJ et al. *JAMA Network Open.*

eTable 22. Associations of Body Mass Index in Women and Men With Odds of Miscarriage, Basic Model

	Women		Men	
	N	OR (95% CI)	N	OR (95% CI)
Body mass index in kg/m^2	2,770	1.02 (1.00-1.05)	2,189	1.00 (0.96-1.04)
Underweight (<18.5)	71	0.99 (0.45-2.20)	17	0.71 (0.09-5.39)
Normal weight (18.5-24.9)	1,688	Reference	1,081	Reference
Overweight (25.0-29.9)	655	1.48 (1.13-1.94)	838	0.98 (0.70-1.37)
Obesity (≥30.0)	356	1.38 (0.97-1.94)	253	0.86 (0.51-1.46)

Values represent the odds of miscarriage per unit increase in and categories of body mass index (95% confidence interval (CI)) from logistic regression models, as compared to the reference category.

© 2024 Boxem AJ et al. *JAMA Network Open.*

eTable 23. Associations of Body Mass Index in Women and Men With Odds of Miscarriage, Adjusted Model

	Women		Men	
	N	OR (95% CI)	N	OR (95% CI)
Body mass index in kg/m^2	2,770	1.02 (1.00-1.05)	2,189	0.99 (0.95-1.03)
Underweight (<18.5)	71	1.01 (0.45-2.27)	17	0.75 (0.10-5.78)
Normal weight (18.5-24.9)	1,688	Reference	1,081	Reference
Overweight (25.0-29.9)	655	1.49 (1.12-1.98)	838	0.90 (0.64-1.27)
Obesity (≥30.0)	356	1.44 (1.00-2.08)	253	0.76 (0.44-1.32)

Values represent the odds of miscarriage per unit increase in and categories of body mass index (95% confidence interval (CI)) from logistic regression models, as compared to the reference category). Models were adjusted for participants age, ethnicity, education level, smoking and alcohol consumption. Parity and history of miscarriage were included in the women's model.

© 2024 Boxem AJ et al. *JAMA Network Open.*

eTable 24. Associations of Body Mass Index in Women and Men With Odds of Miscarriage, Adjusted Model Excluding Couples Undergoing Assisted Reproductive Technology

	Women		Men	
	N	OR (95% CI)	N	OR (95% CI)
Body mass index in kg/m^2	2,506	1.02 (1.00-1.05)	1,998	1.00 (0.95-1.04)
Underweight (<18.5)	60	1.21 (0.53-2.38)	15	0.82 (0.10-6.39)
Normal weight (18.5-24.9)	1,539	Reference	998	Reference
Overweight (25.0-29.9)	583	1.45 (1.08-1.96)	765	0.93 (0.65-1.32)
Obesity (≥30.0)	324	1.52 (1.03-2.22)	220	0.79 (0.44-1.42)

Values represent the odds of miscarriage per unit increase in and categories of body mass index (95% confidence interval (CI)) from logistic regression models, as compared to the reference category. Models were adjusted for participants age, ethnicity, education level, smoking, alcohol consumption, parity and history of miscarriage in the women's model, and stratified for assisted reproductive technology.

© 2024 Boxem AJ et al. *JAMA Network Open.*

eTable 25. Associations of Body Mass Index in Women and Men With Odds of Miscarriage, Adjusted Model Including Only First Episodes

	**Women**		**Men**	
	N	OR (95% CI)	N	OR (95% CI)
Body mass index in kg/m^2	2,483	1.02 (0.99-1.05)	1,965	1.00 (0.95-1.04)
Underweight (<18.5)	65	1.10 (0.49-2.49)	17	0.78 (0.10-6.05)
Normal weight (18.5-24.9)	1,515	Reference	980	Reference
Overweight (25.0-29.9)	580	1.51 (1.12-2.03)	740	0.91 (0.63-1.32)
Obesity (≥30.0)	323	1.39 (0.94-2.05)	228	0.79 (0.44-1.42)

Values represent the odds of miscarriage per unit increase in and categories of body mass index (95% confidence interval (CI)) from logistic regression models, as compared to the reference category. Models were adjusted for participants age, ethnicity, education level, smoking and alcohol consumption. Parity and history of miscarriage were included in the women's model. Only the first episodes were included in the model.

© 2024 Boxem AJ et al. *JAMA Network Open.*

eTable 26. Associations of Body Mass Index in Women and Men With Odds of Miscarriage, Adjusted Model Including Body Mass Index of Partner

	Women		Men	
	N	OR (95% CI)	N	OR (95% CI)
Body mass index in kg/m^2	2,189	1.03 (0.99-1.07)	2,189	0.98 (0.94-1.03)
Underweight (<18.5)	59	1.02 (0.36-2.89)	17	0.74 (0.10-5.76)
Normal weight (18.5-24.9)	1,418	Reference	1,081	Reference
Overweight (25.0-29.9)	472	1.77 (1.23-2.54)	838	0.88 (0.62-1.24)
Obesity (≥30.0)	240	1.43 (0.85-2.41)	253	0.70 (0.40-1.23)

Values represent the odds of miscarriage per unit increase in and categories of body mass index (95% confidence interval (CI)) from logistic regression models, as compared to the reference category. Models were adjusted for body mass index of partner, participants age, ethnicity, education level, smoking and alcohol consumption. Parity and history of miscarriage were included in the women's model.

© 2024 Boxem AJ et al. *JAMA Network Open.*

eTable 27. Associations of Joint Body Mass Index of Women and Men With Odds of Miscarriage, Adjusted Model

**Total population**		
	**Men**	
**Women**	Normal weight (18.5-24.9 kg/m^2)	Overweight and obesity (≥25.0 kg/m^2)
Normal weight (18.5-24.9 kg/m^2)	920 (59)	704 (37)
Overweight and obesity (≥25.0 kg/m^2)	288 (27)	552 (47)
* NA = 1,572 of 4,036 episodes		
**Miscarriage population, stratified for miscarriage**		
	**Men**	
**Women**	Normal weight (18.5-24.9 kg/m^2)	Overweight and obesity (≥25.0 kg/m^2)
Normal weight (18.5-24.9 kg/m^2)	800 (59)	608 (37)
Overweight and obesity (≥25.0 kg/m^2)	242 (27)	464 (47)
* NA = 656 of 2,770 episodes		

	**Women and men combined**	
	Effect Estimate	p-value
Interaction term body mass index women * body mass index men	1.00	0.69
	N	OR (95% CI)
Normal weight in both women and men (18.5-24.9 kg/m^2)	800	Reference
Normal weight in women (18.5-24.9 kg/m^2) * overweight and obesity in men (≥25.0 kg/m^2)	608	0.79 (0.51-1.23)
Overweight and obesity in women (≥25.0 kg/m^2) * normal weight in men (18.5-24.9 kg/m^2)	242	1.49 (0.90-2.45)
Overweight and obesity in both women and men (≥25.0 kg/m^2)	464	1.33 (0.85-2.07)

Values represent the odds of miscarriage (95% confidence interval (CI)) from logistic regression models, as compared to the reference category. Models were adjusted for age, ethnicity, education level, smoking and alcohol consumption of both women and men, and parity and history of miscarriage.

© 2024 Boxem AJ et al. *JAMA Network Open.*
