# Supplementary material for: Preconception and Early-Pregnancy Body Mass Index in Women and Men, Time to Pregnancy, and Risk of Miscarriage
Source: JAMA Netw Open. 2024 Sep 19;7(9):e2436157. doi: 10.1001/jamanetworkopen.2024.36157 (PMC11413718; doi:10.1001/jamanetworkopen.2024.36157)
Supplement: Supplement 2. — Data Sharing Statement [file jamanetwopen-e2436157-s002.pdf]

Data Sharing Statement

Boxem. Preconception and Early-Pregnancy Body Mass Index in Women and Men, Time to Pregnancy, and Risk of Miscarriage. *JAMA Netw Open*. Published September 19, 2024. doi:10.1001/jamanetworkopen.2024.36157

Data
Data available: No

Additional Information
Explanation for why data not available: Data are available upon request to the corresponding author. Unrestricted data sharing is not allowed due to ethical consent and privacy restrictions.
